# Design Principles for Fluid Molecular Ferroelectrics

**DOI:** 10.1002/adma.73977

**Published:** 2026-07-03

**Authors:** Calum J. Gibb, Jordan Hobbs, William C. Ogle, Richard. J. Mandle

**Affiliations:** ^1^ School of Chemistry University of Leeds Leeds UK; ^2^ School of Physics and Astronomy University of Leeds Leeds UK; ^3^ School of Applied Mathematics University of Leeds Leeds UK

**Keywords:** chemical physics, condensed matter physics, ferroelectricity, molecular dynamics, lamellar structure, liquid crystal, materials science, pairing, soft matter, spontaneous polarization

## Abstract

Fluid molecular ferroelectrics are a new class of organic materials where ferroelectricity is found in conjunction with 3D fluidity whilst still retaining spontaneous polarization values comparable to their traditional solid‐state counterparts. One of the major challenges for soft condensed matter physics is predicting whether a fluid molecular material will form ferroelectric phase with nematic or smectic order. Through the synthesis of 45 systematically varied molecules, and by analogy to solid molecular ferroelectrics, it is shown that subtle hydrogen–fluorine (H/F) substitution(s) allows for tuneable *syn*‐parallel pairing motifs resulting in either specific pairings, leading too geometrically constrained lamellar order, or diversified pairings, stabilising nematic ordering. Large‐scale, fully atomistic molecular dynamics simulations reveal that smectic ferroelectricity emerges from discrete lateral pairing modes, whereas nematic phases arise from a multiplicity of equivalent polar configurations. Together, these findings establish experimentally validated design principles for fluid molecular ferroelectrics and provide a predictive framework for engineering functional polar fluids.

## Graphical abstract

A major challenge for soft condensed matter is predicting whether a fluid molecular material will form ferroelectric phase with nematic or smectic order. Through systematic synthesis, and by analogy to solid molecular ferroelectrics, it is shown that subtle hydrogen–fluorine (H/F) substitution(s) allows for tuneable *syn*‐parallel pairing motifs resulting in either specific pairings, leading lamellar order, or diversified pairings, stabilising nematic ordering.

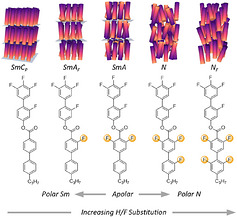

## 1 Introduction

Organic molecular ferroelectric materials find use in emerging technologies such as energy harvesting [1], ultrasonic transducers for medical imaging [2], and actuators in robotics [3]. These solid‐state materials have many advantages over their traditional inorganic counterparts [4, 5] as they can be more lightweight, mechanically flexible and are synthetically more trivial to produce allowing for greater structural diversity [6]. Design driven synthesis of molecular ferroelectrics has advanced significantly and developed several key design mechanisms such as reduced molecular symmetry [7], introduction of homochirality [8], and hydrogen/fluorine (H/F) substitution [9] to tune the desired properties.

Discovered in 2017 [10, 11], fluid molecular ferroelectrics are a new class of organic materials where ferroelectricity is found in conjunction with fluidity (either 3D or 2D depending on the phase) whilst still retaining spontaneous polarization values comparable to their traditional solid‐state counterparts [12, 13, 14, 15, 16, 17, 18]. The addition of fluidity and lack of a true crystal lattice gives fluid ferroelectrics distinct advantages over their solid‐state counterparts (i.e., ease of monodomain fabrication, mechanically stable, structurally diverse), allowing for exploration of new physical phenomena and development of novel applications. As such, a wide variety of potential use cases for fluid ferroelectrics have already been suggested in areas such as quantum optics [19, 20], nanoscale non‐linear electrooptical devices [21, 22, 23, 24], and fluidic capacitors [25].

**FIGURE 1:**
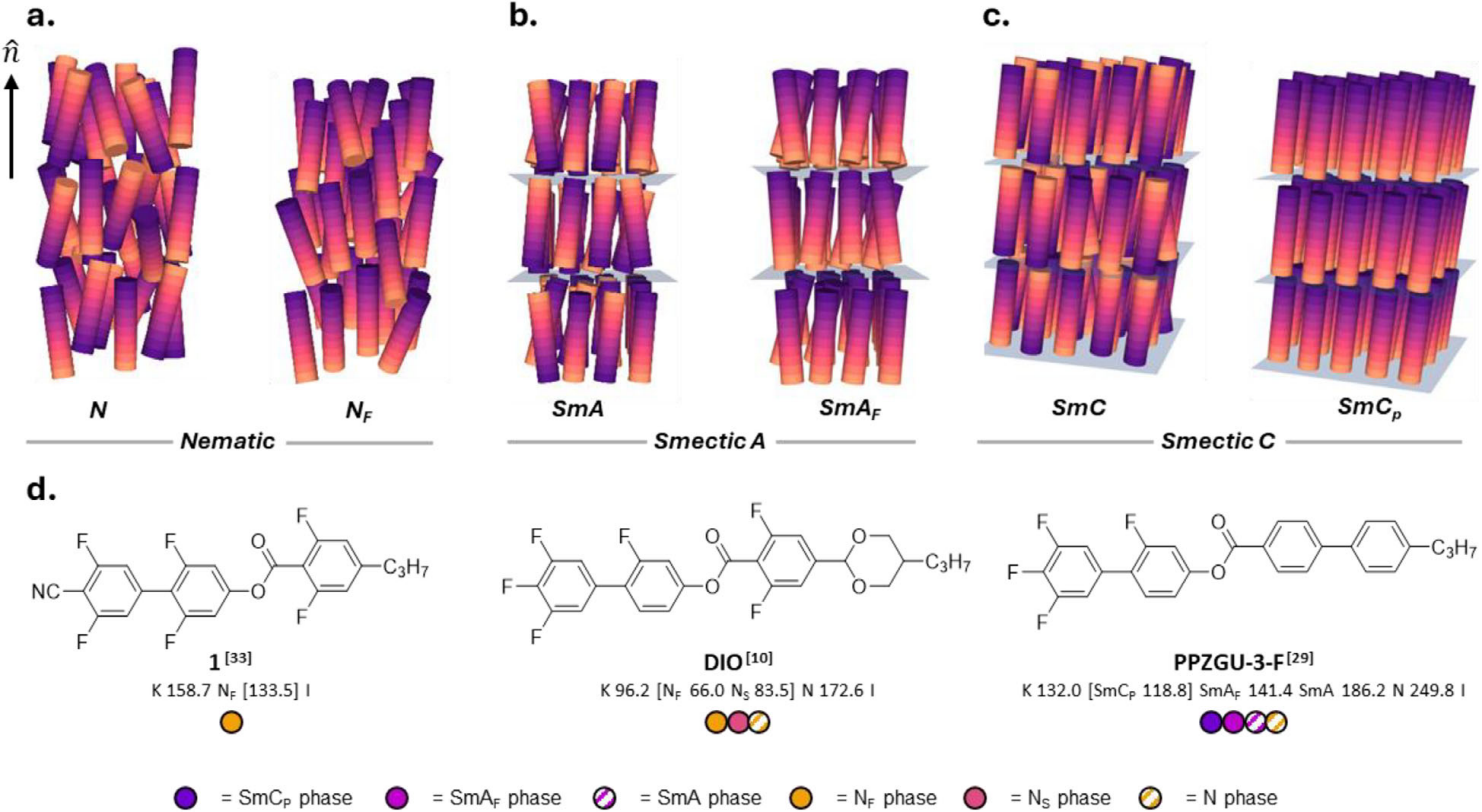
Schematic representations of the para‐ and ferroelectric (a) nematic (N); (b) smectic A (SmA); and (**C**) smectic C (SmC) phases. Each rod is representative of a single molecule with the colour indicating the molecular direction. In fluid molecular ferroelectric phases, the polarisation vector is parallel to the liquid crystalline director ($\hat{n}$). Unlike in their conventional paraelectric analogues, in which the director is symmetry invariant ($\hat{n}$ = ‐$\hat{n}$), the director in a fluid molecular ferroelectric exhibits broken inversion symmetry due to the polarisation ($\hat{n}$ ≠ ‐$\hat{n}$), giving rise to bulk polar order; (d) examples of a typical small molecule fluid ferroelectric materials **1** [33], **DIO** [10] and **PPZGU‐3‐F** [29] with their associated phase profiles and transition temperatures (given in °C) listed below. Striped colours indicate paraelectric phases and solid colours indicate ferroelectric phases. All three molecules possess a rigid, fluorinated aromatic core appended by an EWG (either F or CN) and a short propyl aliphatic chain. Notably **F2100,** which exhibits ferroelectric smectic phases, has a lower degree of H/F substitution compared to **1** and **DIO**.

Fluid molecular ferroelectrics are liquid crystalline, with the majority of materials exhibiting a phase with nematic ordering of its constituents as well as broken inversion symmetry; thus, this phase is termed the ferroelectric nematic, or N_F_, phase (Figure 1a). Further ferroelectric phases have also been discovered such as the ferroelectric smectic A (SmA_F_) [26, 27] (Figure 1b), where the molecules form a one dimensional lamellar structure (manifesting as a density wave) with the molecular long‐axis being orthogonal to the layer normal, and the polar smectic C (SmC_P_) [28, 29, 30] (Figure 1c) where the molecules tilt with respect to layer normal. While the N_F_ phase exhibits 3D fluidity the presence of the layers in the ferroelectric smectics restricts them to only 2D fluidity. As a result, the properties exhibited by the SmA_F_ phase remain quite distinct from the N_F_ phase where the addition of the layers results in stable polarisation memory [31] and deformable soft inclusions [32].

Fluid molecular ferroelectric materials are small organic molecules which currently exist in a constrained area of chemical space. Nominally these consist of a relatively rigid core unit made up of (fluorinated) aromatic rings flanked by a short aliphatic chain at one end and a polar electron withdrawing group (EWG) at the other (Figure 1d). Generally, materials exhibiting such phases have large longitudinal molecular dipole moments (often achieved through H/F substitution) [26, 33, 34, 35], although the relevance of the dipolar magnitude has been debated [36, 37, 38, 39]. Madhusudana [40] suggested that favourable lateral electrostatic interactions along the length of the molecules can promote ferroelectricity with Gibb et al. expanding on this suggesting that uniformly distributed partial dipoles is a further requirement for phase formation [33, 41, 42, 43]. However, moving beyond these postulates to predicting the onset of ferroelectric or indeed the type of phase itself from specific chemical modifications remains an unsolved problem. This makes assigning accurate design principles for the synthesis of fluid ferroelectrics difficult.

At present, there are 438 reported fluid molecular ferroelectrics, 316 exhibit N_F_ order with only around 74 displaying ferroelectricity with lamellar structures. Far less is currently known about the origins of smectic ferroelectricity, although it has been suggested that phase separation of fluorinated and non‐fluorinated aromatic moieties drives layer formation [34, 44]. A singular H/F substitution can be the tipping point between polar nematic and smectic ordering [10, 26, 43, 44] and so the story is reminiscent of solid‐state organic molecular ferroelectrics where such substitution can have a meaningful impact on the thermal stability of ferroelectricity [7]. Despite rapid materials discovery, a predictive framework for ferroelectric phase selection remains elusive. We envisaged that a selective study, whereby the number and position of fluorine atoms across the length of a simple molecule known to exhibit polar smectic order varies, could allow us to tune the delicate balance between nematic and lamellar ferroelectricity, gaining insight into the molecular design of fluid molecular ferroelectrics.

## 2 Results and Discussion

**FIGURE 2:**
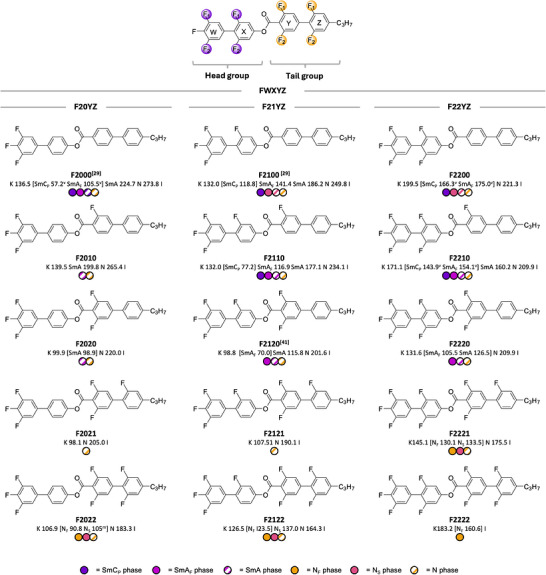
The molecular structures, phase sequences and associated transition temperatures (°C) for the highly fluorinated **FWXYZ** materials. Beginning with the first ring on the head of the molecule, W, X, Y, and Z refer to the number of appended fluorine atoms on each ring and vary between 0 and 2. [ ] indicate a monotropic phase transition, ^m^ indicates a transition temperature determined by POM, and ^V^ is indicative of an extrapolated transition, determined by binary mixture studies between the material and **F2100**. Striped colours indicate apolar phases, and solid colours indicate ferroelectric phases. [Correction added on July 9, 2026, after first online publication: In figure 2 the reference [39] has been changed to [41].]

The initial investigation begins with the synthesis of 15 analogues of a highly fluorinated fluid molecular ferroelectric, whereby the degree of H/F substitution was selectively varied (Figure 2). These materials are named with the acronym **FWXYZ**, whereby F indicates the EWG of the molecule, and W, X, Y, and Z indicate the number of fluorine atoms *ortho* to the EWG or biphenyl linkages and is varied between 0 and 2. The chemical synthesis and structural characterisation of these materials can be found in in the ESI to this article.

**FIGURE 3:**
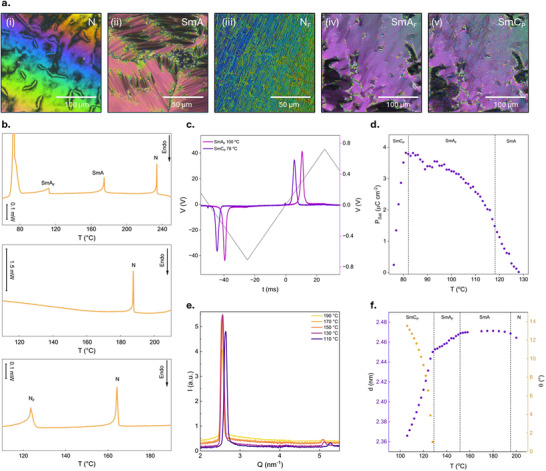
(a) POM micrographs depicting characteristic textures of the (i) N, (ii) SmA, (iii) N_F_, (iv) SmA_F_, and (v) SmC_P_ phases. All images are taken of thin samples sandwiched between untreated glass coverslips; (b) representative DSC thermograms depicting the phase sequence of (top) **F2110**, (middle) **F2121**, (bottom) **F2122**; (c) current response measurement of **F2110** in the SmA_F_ and SmC_P_ phases measured at 10 Hz at 100°C and 78°C, respectively; (d) temperature dependence of the saturated polarization measured for **F2110**; (e) 1D x‐ray scattering pattern measured for **F2100** across its 3 smectic phases. Moving of the peak to larger Q values indicates layer contraction due to molecular tilt; and (f) the temperature dependence of the layer spacing and molecular tilt obtained by SAXS measured for **F2100** in the SmA, SmA_F_ and SmC_P_ phases.

Of the 15 **FWXYZ** analogues, 9 materials exhibit ferroelectricity with the phase‐type (i.e., nematic or smectic) varying with the degree of H/F substitution on the tail‐group of the molecule (Figure 2). Phase sequences of each analogue were determined by polarized optical microscopy (POM; Figure 3a) and differential scanning calorimetry (DSC; Figure 3b). Small angle X‐ray scattering (SAXS) was used to confirm smectic order where implied by POM (Figure 3c,d) while the existence of ferroelectricity was confirmed by a current reversal technique (Figure 3e,f), with a single peak being indicative of a ferroelectric phase. In the case of the SmC_P_ phase, an additional small peak, pre‐voltage polarity reversal is observed which is indicative of the removal of molecular tilt [29, 30]. Several materials did not exhibit polar phases in neat form due to their extreme proclivity for crystallisation. Therefore, extrapolated values for their transition temperatures were found by preparation of binary mixtures with **F2100**, allowing for determination of their phase sequences and associated transitions temperatures (Figure S1–S3). Further data and details to all characterisation techniques is given in the ESI to this article.

Increasing H/F substitution in front of the ester linkage (i.e., the head‐group, Figure 2) results in an increase in the polar‐apolar transition temperatures with a corresponding increase of around 35°C per additional F atom, irrespective of mesophase type observed (i.e., nematic or smectic). This observation is consistent with previous work where similar H/F substitutions have been made [26, 33, 34]. This reflects the resultant stronger lateral core‐core interactions, afforded by the additional H/F substitution, between molecules and a more spatially uniform partial dipole organisation along the long molecular axis. This allows for a better packing of chessboard like electrostatic +ve and ‐ve charge interactions known to be favourable for the formation of fluid ferroelectric phases [33, 45].

Whilst the degree of H/F substitution on the head‐group seems to be vital to the observation of fluid ferroelectricity, fluorination behind the ester (i.e., the tail‐group, Figure 2) has quite a different pattern of effects. When the tail‐group of the molecule has less than 2 appended F atoms (i.e., F < 2), smectic phases are observed, and the thermal stability of these phases decreases with increasing H/F substitution. When the tail‐group possesses more than 2 appended F atoms (i.e., F > 2), N_F_ and N_S_ (splay nematic) [46], an anti‐ferroelectric nematic phase (sometimes referred to as the N_X_ [17, 18] or SmZ_A_ [27] phase) phases are observed with the stability of the phases increasing with increasing H/F substitution. In the intermediate region (i.e., F = 2), only a very low temperature transition to a SmA_F_ phase is observed in **F2220** and **F2120**, with no such phase exhibited by **F2020**. Effectively, this divides the [27] **FWXYZ** analogues into two distinct groups providing us with two general observations. First, H/F substitution of the head‐group has a strong effect on the onset temperature for ferroelectric order while having little to no effect on the phase‐type exhibited by a homologue. Secondly, while H/F substitution of the tail‐group has only a modest effect on the onset temperature of ferroelectricity, it strongly influences molecular packing and dictates the type of phase observed (i.e., nematic or smectic) – this mirrors the behaviour of highly fluorinated apolar liquid crystals, in which fluorination can suppress lamellar organisation [47, 48].

In the case of paraelectric liquid crystal phases, the evolution from smectic to nematic phases with increasing lateral bulk is well understood: increasing lateral fluorination effectively forces molecules apart, decreasing π‐π interactions between the aromatic units and hence destabilising the lamellar structure [49]. In the case of the **FWXYZ** analogues presented here, x‐ray scattering measurements confirm increased lateral spacing upon increasing H/F substitution of the tail‐group (Figure S5), although they also demonstrate that materials that exhibit ferroelectric smectic phases general show stronger π‐π interactions evidenced by a significant shoulder to the wide‐angle scattering peak (Figure S5). It has been suggested that the formation of ferroelectric smectic phases may be in part due to the microphase separation of fluorinated and un‐fluorinated aromatic regions of the molecules (i.e., microphase separation of the front and back of molecules, here that would be the WX and YZ regions [34, 44]).Whilst microphase separation is known to occur when mixing fluorinated and un‐fluorinated aliphatic moieties, the opposite is true for the mixing of their aromatic counterparts [50]. The most obvious example is the favourable interactions which occur between benzene and hexafluorobenzene, where binary mixtures of the two components can be isolated as crystalline solids near to room temperature when the pure material are liquids [51]. We therefore consider the possibility that such microphase separation drives the formation of ferroelectric smectic phases here to be improbable and therefore the molecular origins of lamellar ferroelectricity must be a result of different interactions between the molecules.

**FIGURE 4:**
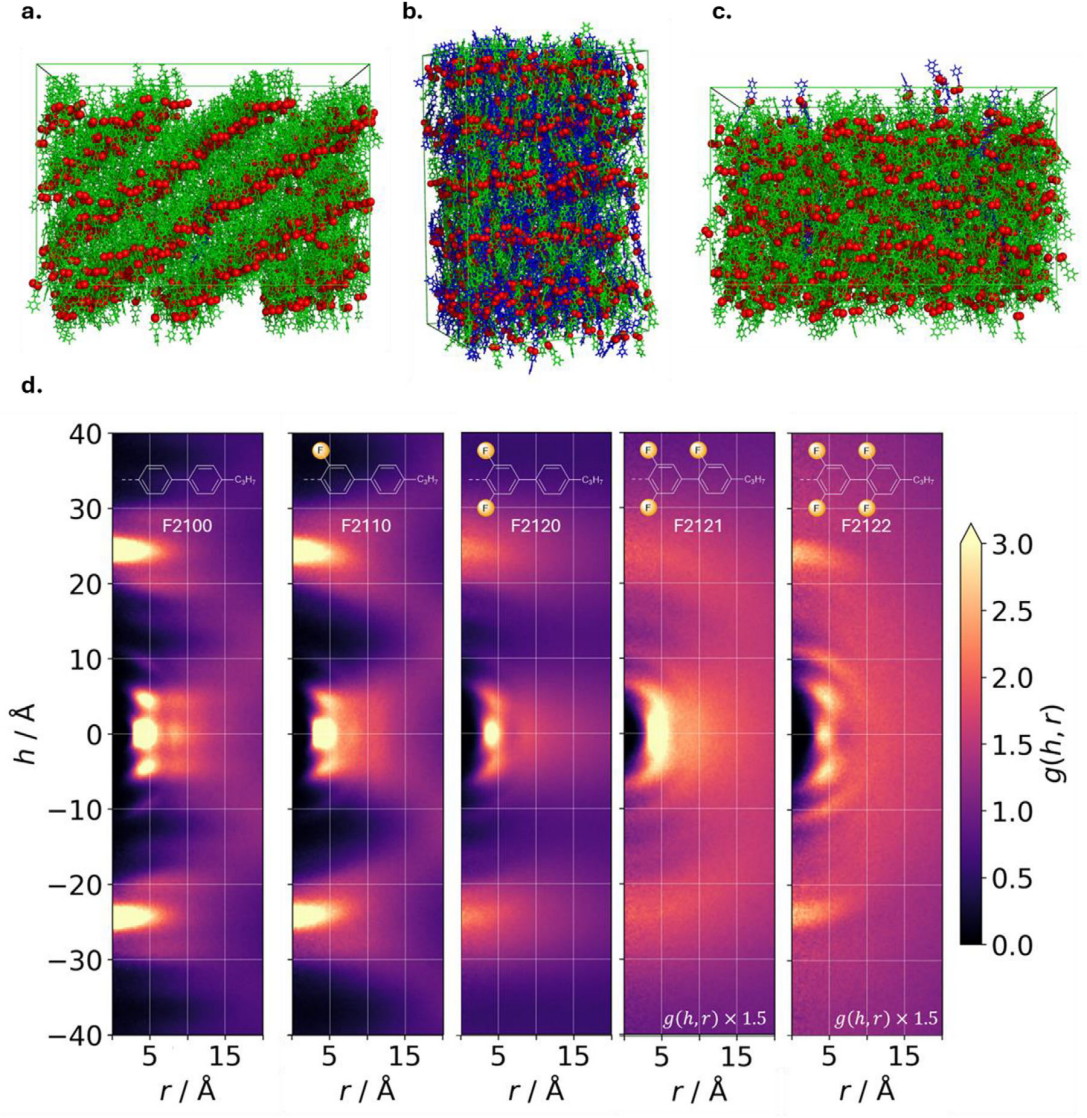
Instantaneous configurations of: (a) the SmC_P_ phase of **F2100**; (b) the apolar SmA phase of **F2120**; (c) the polar N_F_ phase of **F2122**. Molecules aligned parallel to the director are shown as green, molecules shown as antiparallel are shown as blue. The oxygen atoms are shown as spheres to aid visualisation of the layer structure in **F2100** and **F2120** (or its absence in **F2122**); and (d) Cylindrical distribution function (CDF) plots for compounds **F21XY** generated over the final 500 ns of each simulation trajectory.

In‐order to probe the interactions between molecules, we elected to perform large scale, long duration, fully atomistic molecular dynamics (MD) simulations (1000 molecules, > 1 µs) on selected analogues (**F21YZ**, Figure 2 [central column]) with full details of the setup configuration given in the ESI. For the 5 **F21YZ** analogues, each simulation reproduces the same phase type observed experimentally where we see the evolution from ferroelectric smectic to ferroelectric nematic configurations via an intermediate region containing their apolar analogues (Figure 4a–c). **F2100** forms a SmC_P_ phase while **F2110** forms a SmA_F_ phase where the calculated layer spacings and tilt angle (SmC_P_ phase only) are in reasonable agreement with experimental values (Table S3). **F2120** forms an apolar SmA, **F2121** forms an apolar nematic and **F2122** forms a N_F_ phase. In‐order to understand the interactions between the molecules in the different phase types, we compute the cylindrical distribution function (CDF) for the **F21XY** analogues over the final 500 ns of the MD simulations. In the CDF analysis, we use cylindrical binning of the molecular centres‐of‐mass (COM) for all molecules in a simulation over some period of time; preserving information about anisotropic positional order. In its simplest form the CDF (*g*_*h*,*r*_) is:

$$g_{h,r}=\frac{\langle n\left(h,r\right)\rangle }{\rho_{bulk}\times V_{shell}}$$

where 〈*n*(*h*, *r*)〉 is the average count of molecular COM positions in a cylindrical shell centred on the molecular COM of height *h* and radius *r*; normalised against the bulk density of particles (ρ_*bulk*_) in the simulation and cylindrical shell volume (*V_shell_*). In the resulting CDF plot preferred pairing modes show up as high probability regions against the background. Sharp spot‐like features with large *g*_*h*,*r*_ are consistent with positionally specific (and/or likely directional) pairing geometries (e.g. π − π stacking), arc‐like features with more modest *g*_*h*,*r*_ reflect less positional specificity (e.g. dispersion, electrostatic) [52].

Beginning with **F2100**, strong on‐axis features at h = ±24 are observed in the SmC_P_ phase which correspond to head/tail arrangements of molecules in adjacent layers (Figure 4d). The off‐axis features at r ≈ 4.5 correspond to staggered pairs of molecules with face‐face interactions between adjacent 3,4,5‐trifluorobenzene units (features at h ≈ ±4, h ≈  ± 2). There is reasonable in‐plane orientation in the simulated SmC_P_ phase, with the “neighbours neighbour” giving corresponding signals, albeit more diffuse, at r ≈ 9, as well as diffuse areas for head‐tail correlations at h = ±20 and h = ±28. Adding a single fluorine atom to afford **F2110** gives a comparable CDF, albeit more diffuse and with the features at h ≈ ±4, h ≈  ± 2 (staggered parallel pair) notably less prominent. The interaction between staggered 3,4,5‐trifluorobenzene head‐group units on adjacent molecules is significantly perturbed by the introduction of a distal fluorine atom, presumably via repulsion (vide infra). Experimentally, this change in structure is coincident with a 24.5°C reduction in the onset temperature of ferroelectric smectic order and 9.1°C reduction in apolar smectic order indicting the dual effect of reducing inhibiting layers both in the polar and apolar configurations.

For **F2120,** the simulation forms a SmA phase where the features around the molecular length (i.e., h ≈ 23) result from the regular displacement due to the layer structure and are notably weaker and more diffuse than for the SmA_F_ phase of **F2110** as they encode both head/tail, head/head and tail/tail interactions. For the N phase of **F2121,** the diffuse feature around the molecular length (h ≈ 23) reduces in prominence with the strongest feature being a broad lateral separation of molecules (r = 5, h ‐4 and 4). For both **F2120** and **F2121** the sharp features at h ≈ ±4, h ≈  ± 2 (staggered parallel pair) are reduced in prominence. Finally, for **F2122**, which forms an N_F_ phase, shows a qualitatively different CDF dominated by arc‐like intensity features off axis that open out in (h, r) away from the origin. These represent staggered side‐by‐side interactions between molecules, the diffusivity of which indicates a broad distribution of nearest‐neighbour positions without fixed relative geometries. Such behaviour is consistent with other CDF analysis of MD trajectories of other materials exhibiting the N_F_ phase [35, 53]. While the N_F_ phase comprises rather unspecific pairing between molecules (and thus arc‐like g(h,r), Figure 4d), the ferroelectric smectic phases arise from specific geometric pairing motifs (thus spot like features in g(h,r), Figure 4d). Examination of the Cambridge Crystallographic Database (CCDC) reveals that the 3,4,5‐trifluorobenzene motif forms slipped parallel configurations [54]; consistent with the observed g(h,r) for the ferroelectric smectic phases observed for **F2100** to **F2120**.

In other words, the formation of ferroelectric smectic phases is driven, here at least, by specific preferred lateral pairing motifs. As the degree of fluorination begins to increase, these pairings are disrupted by electrostatic repulsion. As the degree of fluorination further increases beyond a certain number (in this case F > 2) the chemical similarity along the molecular length allows for a wider variety of polar pairing modes thus forming polar nematic phases. A logical extension of this is that the high dipoles of the molecules is not necessarily the key driver of ferroelectric smectic phase formation, rather, these could be generated in materials of even modest polarity by careful consideration and engineering of intermolecular interactions.

The balance between the formation of either ferroelectric nematic, ferroelectric smectic or their apolar analogues may then be explained by specific intermolecular pairing modes. Both ferroelectric nematic and smectic phases require a preference for syn‐parallel pairing modes rather than anti‐parallel. If the syn‐parallel pairing has many possible pairing modes, then the phase formed is nematic‐like which occurs for maximally fluorinated tail‐groups (i.e., **FWX22**) as similar F‐π interactions can form along the full length of the molecule. As the number of F atoms decreases and becomes locally segregated along the structure, the number of pairing modes reduces, increasing the tendency toward smectic order due to the reduction in competition. In the intermediate regime (i.e., F = 2) there is neither enough parallel pairing modes along the length to form an N_F_ phase nor sufficient segregation to generate a dominant ferroelectric smectic mode and so apolar behaviour dominates. Experimental evidence strongly suggests competing modes occur in other fluid ferroelectric systems where a transition from an apolar SmA to the N_F_ phase has been observed previously with a sharp change in packing [55]. This observation appears to be general to other fluid ferroelectric systems where H/F substitution is used to drive polar phase behaviour [26, 33, 34, 35, 43, 44].

**FIGURE 5:**
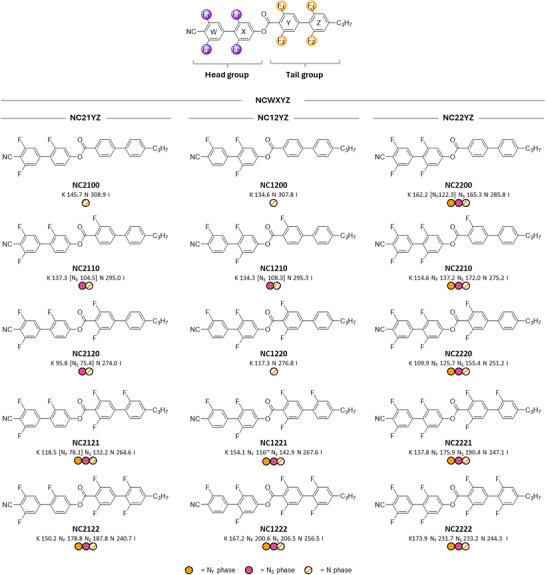
The molecular structures, phase sequences and associated transition temperatures (°C) for the highly fluorinated **NCWXYZ** materials. Beginning with the left most ring on the head‐group of the molecule, W, X, Y, and Z refer to the number of appended fluorine atoms on each ring and vary between 0 and 2. [ ] indicate a monotropic phase transition, ^m^ indicates a transition temperature determined by POM. Striped colours indicate apolar phases, and solid colours indicate polar phases.

To further assess the generality of these observations, another set of analogous materials were synthesised; this time replacing the terminal F atom at the head of the material with a nitrile (CN) moiety. These 30 structures are denoted by **NCWXYZ**. First, let us consider the analogues which possess a minimum of 3 F atoms on the head of the molecule, of which 12 of the materials exhibit fluidic ferroelectricity (Figure 5). In contrast to the **FWXYZ** materials discussed previously, all these **NCWXYZ** homologues display solely nematic ordering with no evolution in phase‐type on increasing H/F substitution on the tail of the molecules.

Similarly to the **FWXYZ** materials, increasing the number of F atoms on the head of the molecule generally sees an increase the onset of polar order with there being only a small difference in the polar transition temperatures between the structural isomers (for example **NC2110** and **NC1210**). The onset of polar order displays a comparable trend to that seen for the FWXYZ materials, whereby increasing from 0 to 2 F atoms on the tail‐group sees the gradual decrease in the polar transition before it begins to increase again for the **NCWX21** and **NCWX22** analogues. Atomistic MD simulations performed on the **NC22YZ** subset are conducted in the N_F_ phase for these materials (Figure S6) and the CDF computed (Figure S7). The CDFs show arc‐like intensity features off axis that open out in (h, r) away from the origin representing staggered side‐by‐side interactions between molecules, the diffusivity of which are consistent with broad distributions of neighbour‐neighbour pairings with minimal fixed geometries (i.e., nematic ordering). These features are consistent for the other **NC22YZ** analogues with increasing H/F substitution having minimal effect of the preferred pairing modes, thus, there is no tendency toward smectic phase formation. These results imply that the terminal nitrile is detrimental to the formation of lamellar fluid ferroelectrics; likely due to its much larger electrostatic presence dominating the molecular interactions. Examination of the literature indicates that this may be a general observation with only 3 of the 74 known ferroelectric smectogens containing a CN moiety [31, 56].

The final 15 **NCWXYZ** materials, in which only a single H atom is substituted for a F atom in the head‐group of the molecule and up to 4 are substituted on the tail‐group (Figure S8), were found to exhibit only paraelectric N and SmA phases. Many of these materials are structural isomers of the materials which exhibit fluid ferroelectricity shown in Figure 5 (for example **NC2200** vs **NC0022**), highlighting the importance of specific positional H/F substitution in the design of fluid molecular ferroelectrics [33]. Fluorination of the head‐group of the molecule has a greater effect on the onset of ferroelectricity, presumably reflecting the enhanced polarity of the head‐group promoting the formation of *syn*‐parallel pairing modes compared to *anti*‐parallel. Further to this, this reinforces the previous observation that microphase segregation of fluorinated and non‐fluorinated areas of the materials does not promote the formation lamellar fluidic ferroelectric phases when there is little fluorination in the head‐group of the molecule compared to the tail‐group.

## 3 Conclusions

Analogous to solid state molecular ferroelectrics [6, 7], H/F substitution has shown to be a powerful and versatile tool for controlling ferroelectric order in fluid molecular systems. In particular, specific fluorination patterns allow not only the thermal stability of the ferroelectric phase to be tuned, but also the nature of the ferroelectric phase itself, determining whether nematic or smectic order is realised.

Across the materials studied, additional H/F substitution at the molecular head group generally enhances ferroelectric phase stability more effectively than the analogous substitution to the tail‐group. However, head‐group fluorination alone does not dictate the type of ferroelectric phase observed. Instead, the balance between nematic and smectic‐like ordering is remarkably delicate, with a single H/F substitution capable of being the tipping point between nematic and smectic behaviour.

MD simulations reveal ferroelectric smectic order arises from specific syn‐parallel pairing between neighbouring molecules, which promote lamellar organisation. These are easily perturbed by additional H/F substitution at the molecular tail, providing a microscopic explanation for the experimentally observed sensitivity of smectic ordering toward subtle changes to molecular structure. The nature of the EWG at the head of the molecule also influences these paring modes whereas a terminal fluorine can be efficacious toward the formation of ferroelectric smectic phases as well as nematics. Nitriles in this study are solely nematogenic. This dependency maybe easily observed by computing the CDF from MD simulation and hence could be used to probe other EWG.

This study provides a set of design principles and a predictive framework which, when taken together, can inform the design of future fluid molecular ferroelectrics with specific properties and, for the first time, these can be tailored toward specific practical applications. Our results show that ferroelectric ordering in fluids is governed less by dipolar magnitude and more by the geometry of accessible pairing modes, reframing the molecular origins of spontaneous polar ordering in soft matter systems. The presented results mark the transition from empirical discovery of fluid ferroelectric materials toward predictive, rational molecular design.

## Author Contributions

C.J.G., W.C.O., and R.J.M. performed chemical synthesis, purification and structural characterisation. C.J.G performed mixture formulation and analysis. J.H and C.J.G. performed optical microscopy and calorimetry measurements. J.H. performed SAXS/WAXS and applied field studies. R.J.M. performed MD simulations and electronic structure calculations. All authors contributed to the conception of this work, to the interpretation of results, and to writing of the manuscript.

## Conflicts of Interest

The authors declare no conflicts of interest.

## Supporting information




**Supporting File**: adma73977‐sup‐0001‐SuppMat.docx.

## Data Availability

The data associated with this paper are openly available from the University of Leeds Data Repository at https://doi.org/10.5518/1792.
