# Supplementary material for: Design Principles for Fluid Molecular Ferroelectrics
Source: Adv Mater. 2026 Jul 3;38(44):e73977. doi: 10.1002/adma.73977 (PMC13449159; doi:10.1002/adma.73977)
Supplement: Supplementary file 1 [file ADMA-38-e73977-s001.docx]

Design Principles for Fluid Molecular Ferroelectrics

Supplemental Information

Calum J. Gibb^1^, Jordan Hobbs^2^, William Ogle^1,3^, Richard. J. Mandle*^1,2^

^1^ School of Chemistry, University of Leeds, Leeds, UK, LS2 9JT.

^2^ School of Physics and Astronomy, University of Leeds, Leeds, UK, LS2 9JT.
^3^_­_ School of Applied Mathematics, University of Leeds, Leeds, UK, LS29JT.

*Author for correspondence e-mail: r.mandle@leeds.ac.uk

**Contents**

1 **Supplementary Methods**
  1.1 Chemical synthesis
  1.2 Chemical characterisation methods
  1.3 Mesophase characterisation
  1.4 X-ray scattering
  1.5 Current response measurements
  1.6 DFT calculations
  1.7 Molecular Dynamics
2 **Supplemental results**
3 **Organic Synthesis**
4 **Supplemental References**

**1	Supplementary Methods**

**1.1	Chemical Synthesis**

Chemicals were purchased from commercial suppliers (Fluorochem, Merck, Apollo Scientific) and used as received. Solvents were purchased from Merck and used without further purification. Reactions were performed in standard laboratory glassware at ambient temperature and atmosphere and were monitored by TLC with an appropriate eluent and visualised with 254 nm or 365 nm light. Chromatographic purification was performed using a Combiflash NextGen 300+ System (Teledyne Isco) with a silica gel stationary phase and a hexane/ethyl acetate gradient as the mobile phase, with detection made in the 200-800 nm range. Chromatographed materials subjected to re-crystallisation from an appropriate solvent system.

**1.2**	**Chemical Characterisation Methods**

The structures of intermediates and final products were determined using ^1^H, ^13^C{^1^H} and ^19^F NMR spectroscopy. NMR spectroscopy was performed using either a Bruker Avance III HDNMR spectrometer operating at 400 MHz, 100.5 MHz or 376.4 MHz (^1^H, ^13^C{^1^H} and ^19^F, respectively) or a Bruker AV4 NEO 11.75T spectrometer operating at 500 MHz or 125.5 MHz (^1^H and ^13^C{^1^H}, respectively). Unless otherwise stated, spectra were acquired as solutions in deuterated chloroform, coupling constants are quoted in Hz, and chemical shifts are quoted in ppm.

**1.3**	**Mesophase Characterisation**

Transition temperatures and measurement of associated latent heats were measured by differential scanning calorimetry (DSC) using a TA instruments Q2000 heat flux calorimeter with a liquid nitrogen cooling system for temperature control. Between 3-8 mg of sample was placed into T-zero aluminium DSC pans and then sealed. Samples were measured under a nitrogen atmosphere with 10 °C min^-1^ heating and cooling rates. The transition temperatures and enthalpy values reported are averages obtained for duplicate runs. In general LC phase transition temperatures are measured on cooling from the onset of the transition while melt temperatures were measured on heating to avoid crystallization loops that can occur on cooling. Phase identification by polarised optical microscopy (POM) was performed using a Leica DM 2700 P polarised optical microscope equipped with a Linkam TMS 92 heating stage. Samples were studied sandwiched between two untreated glass coverslips.

**1.4	X-ray Scattering**

X-ray scattering measurements, both small angle (SAXS) and wide angle (WAXS) were recorded using an Anton Paar SAXSpoint 5.0. This was equipped with a primux 100 Cu X-ray source with a 2D EIGER2 R detector with a variable sample-detector distance. The X-rays had a wavelength of 0.154 nm. Samples were filled into either thin-walled quartz capillaries or held between Kapton tape. Temperature was controlled using an Anton Paar heated sampler with a range of 20◦C to 300◦C and the samples held in a chamber with an atmospheric pressure of >1 mbar. Background scattering patterns were recorded, scaled according to the sample transmission and then subtracted from the samples’ obtained 2D SAXS pattern. 1D patterns were obtained by radially integrating the 2D SAXS patterns. Peak positions and FWHM were recorded and then converted into d spacing following Bragg’s law. In tilted smectic phases, the tilt was obtained from:

$$\frac{d_{C}}{d_{A}}=cos \theta$$

where d_C_ is the layer spacing in the tilted smectic phase, d_A_ is the extrapolated spacing from the non-tilted preceding phase, extrapolated to account for the weak temperature dependence of the preceding phases due to shifts in conformation and order, and θ the structural tilt angle.

**1.5**	**Current Response Measurements**

Current Response measurements are undertaken using the current reversal technique. Triangular waveform AC voltages are applied to the sample cells with an Agilent 33220A signal generator (Keysight Technologies), and the resulting current outflow is passed through a current-to-voltage amplifier and recorded on a RIGOL DHO4204 high-resolution oscilloscope (Telonic Instruments Ltd, UK). Heating and cooling of the samples during these measurements is achieved with an Instec HCS402 hot stage controlled to 10 mK stability by an Instec mK1000 temperature controller. The LC samples are held in 4μm thick cells with no alignment layer, supplied by Instec. The measurements consist of cooling the sample at a rate of 1 K min^-1^ and applying a set voltage at a frequency of 20 Hz to the sample every 1 K. The voltage was set such that it would saturate the measured PS and was determined before final data collection. There are three contributions to the measured current trace: accumulation of charge in the cell (I_c_), ion flow (I_i_), and the current flow due to polarisation reversal (I_p_). To obtain a P_Sat_ value, we extract the latter, which manifests as one or multiple peaks in the current flow, and integrate as:

$$P_{Sat}=\int\frac{I_{p}}{2A}dt$$

where A is the active electrode area of the sample cell.

**1.6**	**DFT Calculations**

Electronic structure calculations were performed using Gaussian G16 revision C.02 ^1^ and with a B3LYP-GD3BJ/cc-pVTZ ^2-4^ basis set. Obtained structures were verified as a minimum from frequency calculations.

**1.7	Molecular Dynamics**

Parameters for the **F21YZ** and **NC22YZ** were modelled using the GAFF forcefield ^5^ with modifications for liquid crystalline molecules ^6,7^. The initial geometries of compounds **F21YZ** and **NC22YZ** were obtained by optimisation (at the B3LYP-GD3BJ/cc-pVTZ level of DFT, as above). RESP charges were calculated at the HF/6-31G* levelClick or tap here to enter text. ^8^ using Gaussian G16.c02 ^1^ with the route section “iop(6/33=2) iop(6/42=6) pop(chelpg, regular)“. Using Antechamber (part of AmberTools24 software package ^9^) the Gaussian output file was converted to mol2 format; finally, this was parsed into Gromacs readable .itp format using the ACPYPE script ^10^. Simulations were performed in Gromacs 2024.4 ^11^ using GPU acceleration (Nvidia L40s) *via* CUDA 13.6.2; bonded, non-bonded, PME and update tasks were offloaded to the GPU during the production run.

Initial coordinates were generated *pseudo* randomly using the Gromacs tool *gmx insert-molecules*; we construct a low-density (~ 0.1 g cm^3^) initial configuration consisting of 1000 molecules with random positional and orientational order. To this we then perform energy minimisation *via* steepest descent, followed by equilibration in the NVE and NVT regimes (the later at T=600K). A brief (20 ns) compression simulation with an isotropic barostat (*P* = 100 Bar) at T=600K affords a liquid-like density isotropic state, used as a starting configuration for subsequent simulations. In the case of polar simulations, a static electric field was applied across the x-axis of the simulation for 50 ns (at T=600K) to give a near saturated polar order parameter (<*P*1> > 0.95). This polar configuration was used as a starting point for all subsequent simulations which were performed at with an anisotropic barostat (P = 1 Bar, allowing the simulation box to deform). For simulations of the **FWXYZ** materials the simulation temperature was 373 K; for the **NCWXYZ** materials the temperature chosen was 25 K below the experimental ferroelectric onset temperature.

For each production MD simulation, we employ a total simulation time of 1 microsecond once stable values of <P1> and <P2> are achieved.

Simulations employed periodic boundary conditions in xyz. Bonds lengths were constrained to their equilibrium values with the LINCS algorithm ^12^. Compressabilities in xyz dimensions were set to 4.5e-5, with the off-diagonal compressibilities were set to zero to ensure the simulation box remained rectangular. Long-range electrostatic interactions were calculated using the Particle Mesh Ewald method with a cut-off value of 1.2 nm. A van der Waals cut-off of 1.2 nm was used. A timestep of 2 fs was employed in all cases. Pressure coupling was via a Parinello-Rahman barostat, and temperature coupling via a velocity rescaling thermostat.

A simulation was considered liquid crystalline if the second-rank orientational order parameter (<*P*2>), calculated *via* the eigenvalues of the nematic Q-tensor using MdTraj v1.9.8^13^ , was greater than 0.3; conversely, the simulation was judged to be isotropic if <*P2*> was below this value). The polar order parameter (<*P*1>) is found as the quotient of the total simulation dipole moment and the maximum possible dipole moment of the simulation^14^. Phase assignment was made by visual inspection of the trajectory. Cylindrical distribution functions were calculated using the cylindr program ^15^. Analysis was conducted on each trajectory frame, with presented values are given as the average over all frames in the final 500 ns of the production MD simulation.

**2	Supplemental Results**

**Table S1.**	Tabulated phase sequences, including transition temperatures (°C) and associated enthalpies of transition (KJ mol^-1^) for the **FWXYZ** materials. Data for **F2000**, **F2100**, and **F2120** have been published previously ^16,17^.

| Material | Phase sequence | |
| --- | --- | --- |
|  | Transition Temperatures (°C) | Enthalpies (KJ mol^-1^) |
| **F2010** | K 139.5 SmA 199.8 N 256.4 I | K 29.5 SmA 0.33 N 0.26 I |
| **F2020** | K 99.9 [SmA 98.9] N 220.0 I | K 24.7 [SmA 0.06] N 0.58 I |
| **F2021** | K 98.1 N 205.0 I | K 27.3 N 0.27 I |
| **F2022** | K 106.9 [N_F_ 90.8 N_S_ 105^m^] N 182.3 I | K 25.7 [N_F_ 25.7 N_S_ 105^m^] N 0.43 I |
| **F2110** | K 91 [SmC_P_ 77.2] SmA_F_ 116.9 SmA 177 .1 N 234.1 I | K 19.5 [SmC_P_ 0.002] SmA_F_ 0.69 SmA 0.30 N 0.39 I |
| **F2121** | K 107.5 N 190.1 I | K 30.7 N 0.32 I |
| **F2122** | K 126.5 [N_F_ 123.5] N_S_ 137^m^ N 164.3 I | K 26.2 [N_F_ 26.2] N_S_ (N/A) N 0.36 I |
| **F2200** | K 199.5 [SmC_P_ 166.3^V^ SmA_F_ 175.0^V^] N 221.3 I | K 28.5 [SmC_P_ (N/A) SmA_F_ (N/A)] N 0.22 I |
| **F2210** | K 171.1 [SmC_P_ 143.9^V^ SmA_F_ 153.9^V^ SmA 160.2] N 209.9 I | K 26.0 [SmC_P_ (N/A) SmA_F_ (N/A) SmA 0.28] N 0.22 I |
| **F2220** | K 131.6 [SmA_F_ 105.5 SmA 126.5] N 182.1 I | K 33.6 [SmA_F_ 0.09 SmA 0.30] N 0.31 I |
| **F2221** | K 145.1 [N_F_ 130.1 N_S_ 133.5] N 175.5 I | K 33.9 [N_F_ 0.25 N_S_ 0.002] N 0.30 I |
| **F2222** | K 183.2 N_F_ 160.6 I | K 43.6 N_F_ 3.65 I |

**Table S2.**	Tabulated phase sequences, including transition temperatures (°C) and associated enthalpies of transition (KJ mol^-1^) for the **NCWXYZ** materials

| Material | Phase sequence | |
| --- | --- | --- |
|  | Transition Temperatures (°C) | Enthalpies (KJ mol^-1^) |
| **NC2100** | K 145.7 N 308.9 I | K 39.1 N 1.40 I |
| **NC2110** | K 137.3 [N_S_ 104.5] N 295.0 I | K 38.0 [N_S_ 0.02] N 1.14 I |
| **NC2120** | K 95.8 [N_S_ 75.4] N 274.0 I | K 22.7 [N_S_ 0.01] N 0.56 I |
| **NC2121** | K 118.5 [N_F_ 78.1] N_S_ 132.2 N 264.6 I | K 31.3 [N_F_ 0.11] N_S_ 0.01 N 0.61 I |
| **NC2122** | K 150.2 N_F_ 178.8 N_S_ 187.8 N 240.7 I | K 36.3 N_F_ 0.23 N_S_ 0.01 N 0.65 I |
| **NC1200** | K 134.6 N 307.8 I | K 31.2 N 1.90 I |
| **NC1210** | K 134.3 [N_S_ 108.3] N 295.3 I | K 41.2 [N_S_ 0.02] N 1.74 I |
| **NC1220** | K 117.3 N 276.8 I | K 35.1 N 1.62 I |
| **NC1221** | K 154.1 [N_F_ 116^m^ N_S_ 142.9] N 267.6 I | K 40.5 [N_F_ N/A N_S_ 0.010] N 0.97 I |
| **NC1222** | K 167.2 N_F_ 200.6 N_S_ 206.5 N 256.5 I | K 167.2 N_F_ 200.6 N_S_ 206.5 N 256.5 I |
| **NC2200** | K 162.2 [N_F_ 122.3] N_S_ 165.3 N 285.8 I | K 43.7 [N_F_ 0.30] N_S_ 0.011 N 2.03 I |
| **NC2210** | K 114.6 N_F_ 137.2 N_S_ 172.0 N 275.2 I | K 29.0 N_F_ 0.64 N_S_ 0.024 N 0.67 I |
| **NC2220** | K 109.9 N_F_ 125.7 N_S_ 155.4 N 251.2 I | K 29.4 N_F_ 0.36 N_S_ 0.014 N 0.60 I |
| **NC2221** | K 137.8 N_F_ 175.9 N_S_ 190.4 N 247.1 I | K 36.9 N_F_ 0.41 N_S_ 0.0.22 N 1.37 I |
| **NC2222** | K 173.9 N_F_ 231.7 N_S_ 233.2 N 244.3 I | K 139.1 N_F_ 0.78 N_S_ 0.017 N 1.84 I |

**Fig. S1.**	Binary mixture diagram between **F2200** and **F2100**. The dashed line indicates the crystallisation temperature obtained on cooling from the relevant DSC thermograms.

**Fig. S2.**	Binary mixture diagram between **F2210** and **F2100**. The dashed line indicates the crystallisation temperature obtained on cooling from the relevant DSC thermograms.

**Fig. S3.**	Binary mixture diagram between **F2000** and **F2100**. The dashed line indicates the crystallisation temperature obtained on cooling from the relevant DSC thermograms.

**Fig. S4.**	Current response measurements for **F2110**, **F2220**, **F2221** and **F2222.** All were taken using a 10 Hz triangular waveform with the maximum voltage as marked on the graphs.

**Fig. S5.**	WAXS patterns for **a.** **F2100** and **b.** **F2000** showing how the peak evolves with temperature. At around 19 nm^-1^ a second peak can be observed that increases in intensity at lower temps indicating an increase in π-π stacking interactions. This is more prominent for **F2100** than for **F2000** due to its increase proclivity for ferroelectric phase formation. **c.** Temperature dependence of the lateral spacing for the molecules shown. The value seems to be a function of the number of fluorine substitutions irrespective of fluorination position.

**Table S3.**	Tabulated data from molecular dynamics simulations of compounds **F21YZ** at 373 K. Given values are the average over the final 500 ns of the production MD simulation, with uncertainties given as one standard deviation. ^#^ Nonpolar phase as evidenced by <P1> and visualisation.

| **Material** | **Phase** | $\boldsymbol{\rho}$ **/ g cm^3^** | **<P1>** | **<P2>** | $\boldsymbol{\tau}$ | **d / nm**  **(exp.)** | $\boldsymbol{\theta/ ^{\circ}}$  **(exp.)** |
| --- | --- | --- | --- | --- | --- | --- | --- |
| **F2100** | SmC_P_ | 1.245  $\pm$0.003 | 0.93 $\pm$0.01 | 0.82  $\pm$0.04 | 0.39  $\pm$ 0.05 | 2.1$\pm$0.1 | 30.5$\pm$9.4 |
| **F2110** | SmC_P_ | 1.273  $\pm$0.003 | 0.91 $\pm$0.01 | 0.81  $\pm$0.06 | 0.45  $\pm$ 0.05 | 2.1$\pm$0.2 | 30.5$\pm$10.7 |
| **F2120** | SmA^#^ | 1.269  $\pm$0.005 | 0.04  $\pm$0.02 | 0.72  $\pm$0.10 | 0.25  $\pm$ 0.03 | 2.5$\pm$0.4 | n/a |
| **F2121** | N^#^ | 1.297  $\pm$0.004 | 0.05  $\pm$0.04 | 0.62  $\pm$0.04 | 0.06  $\pm$ 0.04 | n/a | n/a |
| **F2222** | N_F_ | 1.337  $\pm$0.004 | 0.89  $\pm$0.03 | 0.76  $\pm$0.03 | 0.08  $\pm$ 0.04 | n/a | n/a |

**Fig. S6.**	Instantaneous configurations of **NC2200** in the polar N_F_ phase at 383 K. Molecules aligned parallel to the director are shown as green. The nitrogen atoms are shown as spheres to aid visualisation.

**Fig. S7.**	Cylindrical distribution function (CDF) plots for **NC2200**, **F2200**, and **F2210** generated over the final 500 ns of each simulation trajectory.

**Fig. S8.**	The molecular structures, phase sequences and associated transition temperatures (°C) for the fluorinated **NCWXYZ** materials. Beginning with the first ring on the head of the molecule, W, X, Y, and Z refer to the number of appended fluorine atoms on each ring and vary between 0 and 2. [ ] indicate a monotropic phase transition, ^d^ indicates sample decomposition on reaching the isotropic phase on the first heating cycle. Striped colours indicate paraelectric phases.

3	**Organic synthesis**

The total synthesis of materials **FWXYZ** and **NCWXYZ** is outlined in **Scheme S1**. The synthesis of *3,5-difluoro-4'-propyl-[1,1'-biphenyl]-4-carboxylic acid* is reported elsewhere ^16^. The synthesis of the various phenolic starting materials has also been described previously within the literature ^16,18,19^.

**Scheme S1.**	General esterification procedure for the synthesis of the **FWXYZ** and **NCWXYZ** materials.

**3.1	Preparation of *4′-propyl-3-fluorobiphenyl-4-carboxylic acid***

The preparation of *4′-propyl-3-fluorobiphenyl-4-carboxylic acid* is described in **Scheme S2**. A reaction flask was charged with 4*-propylphenyl boronic acid* (5.41 g, 33 mmol) and dissolved in 200 mL of THF and 50 mL of 2M aqueous K_2_CO_3_. The resultant solution was sparged with N_2)_ for 30 mins. *4-bromo-2-fluorobenzoic acid* (6.57 g, 30 mmol) was then added to the sparged solution which was then heated to 70 ℃ (external temperature) under reflux and under an atmosphere of dry nitrogen gas. Once at temperature, PdXPhos (G3) (0.05 mol %) was then added in a single portion and the reaction left for 18 h with constant stirring. The reaction was then cooled, acidifed using 2M HCl and extracted with EtOAc, the aqueous and organic layers separated, the organics being dried over MgSO_4_. Removal of volatiles *in vacuo* afforded an off white solid which was re-crystalised from MeCN to afford the title compound as a fine, white powder.

**Scheme S2**.	Synthesis of *4′-propyl-3-fluorobiphenyl-4-carboxylic acid*

*4′-propyl-3-fluorobiphenyl-4-carboxylic acid*

Yield:	(fine white powder) 7.1 g, 91%

R_F_ (EtOAc):	0.25

^1^H NMR (501 MHz, DMSO):	13.21 (s, 1H, Ar-COO**H**), 7.92 (t, *J* = 8.2 Hz, 1H, Ar-**H**), 7.69 (ddd, *J* = 8.2, 2.2, 1.8 Hz, 2H, Ar-**H**), 7.65 – 7.58 (m, 2H, Ar-**H**)*, 7.31 (ddd, *J* = 8.4, 2.0, 1.7 Hz, 2H, Ar-**H**), .61 (t, *J* = 7.4 Hz, 2H, Ar-C**H_2_**-CH_2_), 1.62 (h, *J* = 7.2 Hz, 2H, CH_2_-C**H_2_**-CH_3_), 0.91 (t, *J* = 7.3 Hz, 3H, CH_2_-C**H_3_**) (*Overlapping signals).

^13^C{^1^H} NMR (101 MHz, DMSO):	165.33 (d, *J* = 3.2 Hz), 162.16 (d, *J* = 256.8 Hz), 146.88 (d, *J* = 8.7 Hz), 143.60, 135.46, 132.98, 129.56, 122.59 (d, *J* = 3.2 Hz), 117.91 (d, *J* = 10.3 Hz), 114.92 (d, *J* = 23.3 Hz), 37.33, 24.40, 14.09.

^19^F NMR (376 MHz, DMSO):	-109.81 (t, *J_F-H_* = 8.2 Hz, 1F, Ar-**F**).

**3.2	Preparation of *2',3,5-trifluoro-4'-propyl-[1,1'-biphenyl]-4-carboxylic acid***

The preparation of *2',3,5-trifluoro-4'-propyl-[1,1'-biphenyl]-4-carboxylic acid* is described in **Scheme S3**.

**Scheme S3.**	Preparation of *2',3,5-trifluoro-4'-propyl-[1,1'-biphenyl]-4-carboxylic acid*

**3.2.1. Synthesis of *4-chloro-2,3',5'-trifluoro-1,1'-biphenyl***

A reaction flask was charged with *4-chloro, 2-fluro bromo benzene* (21.0 g, 100 mmol) and dissolved in 250 mL of THF and 60 mL of 2M aqueous K_2_CO_3_. The resultant solution was sparged with N_2(g)_ for 30 mins. *2, 6-difluro benzene boronic acid* (17.4 g, 110 mmol) was then added to the sparged solution which was then heated to 70 ℃ (external temperature) under an atmosphere of dry nitrogen. Once at temperature, PdXPhos (G3) (0.05 mol %) was then added in a single portion and the reaction left for 18 h with constant stirring. The reaction was then cooled, extracted with EtOAc, the aqueous and organic layers separated, the organics being dried over MgSO_4._ The suspension was filtered, concentrated *in vacuo* and purified by flash chromatography over silica gel using an isocratic run of hexane using a Combiflash NextGen300+ system. The resulting solid was then re-crystallised from hexane:toluene (10:1) to yield 4-chloro-2,3',5'-trifluoro-1,1'-biphenyl as a white, crystalline solid.

*4-chloro-2,3',5'-trifluoro-1,1'-biphenyl*

Yield:	(white solid) 16.5 g, 68 %

R_F_ (Hexanes):	0.33

^1^H NMR (400 MHz):	7.39 (t, *J* = 8.3 Hz, 1H, Ar-**H**), 7.30 – 7.22 (m, 2H, Ar-**H**) ^†^, 7.15 – 7.04 (m, 2H, Ar-**H**), 6.88 (tt, *J* = 8.9, 2.3 Hz, 1H, Ar-**H**). (^†^ Overlapping CDCl_3_).

^19^F NMR (376 MHz):	-109.44 (t, *J_F-H_* = 7.9 Hz, 2F, Ar-**F**), -114.59 (t, *J_F-H_* = 9.4 Hz, 1F, Ar-**F**).

**3.2.2. Synthesis of *2,3',5'-trifluoro-4-propyl-1,1'-biphenyl***

In a flame dried flask, *4-chloro-2,3',5'-trifluoro-1,1'-biphenyl* (11.0 g, 45 mmol) and Fe(acac)_3_ (g, mmol) were dissolved in 250 mL dry THF:NMP (10:1). The solution was cooled over an ice bath before a solution of freshly prepared Grignard reagent (50 mmol) in dry Et_2_O was added dropwise to the stirred solution. The progress of the reaction was followed by ^1^H NMR with no further reaction occurring after 1.5 h. The solution was quenched with 2M HCl (crica. 50 mL) before being extracted with EtOAc, dried over MgSO_4_ and a yellow oil was retrieved under reduced pressure. The oil was found to be a mixture of the solid starting material and the desired product. To purify the mixture, the remaining starting material was crystallized out of solution using hexanes and removed by filtration**.** The filtrate was concentrated reduced pressure to give *2,3',5'-trifluoro-4-propyl-1,1'-biphenyl* as a colourless oil.

*2,3',5'-trifluoro-4-propyl-1,1'-biphenyl*

Yield:	(colourless oil) 11.4 g, 91 %

R_F_ (Hexanes):	0.33

^1^H NMR (400 MHz):	7.31 (t, *J* = 8.1 Hz, 1H, Ar-**H**), 7.12 – 6.96 (m, 4H, Ar-**H**)*, 6.80 (tt, *J* = 9.0, 2.3 Hz, 1H, Ar-**H**), 2.63 (t, *J* = 7.6 Hz, 2H, Ar-C**H_2_**-CH_2_), 1.68 (h, *J* = 7.6 Hz, 2H,m CH_2_-CH_2_-CH_3_), 0.98 (t, *J* = 7.4 Hz, 3H, CH_2_-C**H_3_**). ^*^Overlapping Signals.

^19^F NMR (376 MHz):	-110.23 (t, *J_F-H_* = 8.2 Hz, 2F, Ar-**F**), -118.30 (t, *J_F-H_* = 9.9 Hz, 1F, Ar-**F**).

**3.2.3	Preparation of *2',3,5-trifluoro-4'-propyl-[1,1'-biphenyl]-4-carboxylic acid***

A solution of *n-butyl lithium* (1.6 M in hexanes, 45 mmol, 28 mL) was added dropwise to a stirred, cooled (-78 °C) solution of *2,3',5'-trifluoro-4-propyl-1,1'-biphenyl* (10.0 g, 40 mmol) in anhydrous THF (150 mL) under an atmosphere of dry nitrogen. The aryl lithium solution was allowed to stir for 1.5 h at this same temperature before adding a large excess of gaseous carbon dioxide which was pre-dried by bubbling through conc. H_2_SO_4_. The solution was then allowed too slowly warm to ambient temperature (~ 2 h). The basic solution was acidified with 2M HCl (~100 ml) and extracted with EtOAc. The organics were then dried over MgSO_4_ and a white solid retrieved under reduced pressure. These were then purified by recrystallization from MeCN to give *2',3,5-trifluoro-4'-propyl-[1,1'-biphenyl]-4-carboxylic acid* as a fine white solid.

*2',3,5-trifluoro-4'-propyl-[1,1'-biphenyl]-4-carboxylic acid*

Yield:	(fine white solid) 10.1 g, 86 %

R_F_ (DCM):	0.00

^1^H NMR (400 MHz, DMSO):	7.54 (t, *J* = 8.2 Hz, 1H, Ar-**H**), 7.40 (ddd, *J* = 9.4, 4.6, 2.8 Hz, 2H, Ar-**H**), 7.23 – 7.13 (m, 2H, Ar-**H**)^*^, 2.61 (t, *J* = 7.5 Hz, 2H, Ar-C**H_2_**-CH_2_), 1.61 (h, *J* = 7.3 Hz, 2H, CH_2_-C**H_2_**-CH_3_), 0.90 (t, *J* = 7.3 Hz, 3H, CH_2_-C**H_3_**). *Overlapping signals.

^19^F NMR (376 MHz, DMSO):	-111.81 (d, *J_F-H_* = 10.0 Hz, 2F, Ar-**F**), -118.21 (t, *J_F-H_* = 10.0 Hz, 1F, Ar-**F**).

**3.3	Preparation of *2',3,5,6'-tetrafluoro-4'-propyl-[1,1'-biphenyl]-4-carboxylic acid***

The preparation of *2',3,5,6'-tetrafluoro-4'-propyl-[1,1'-biphenyl]-4-carboxylic acid* is described in **Scheme S4**.

**Scheme S4.**	Preparation of *2',3,5,6'-tetrafluoro-4'-propyl-[1,1'-biphenyl]-4-carboxylic acid*.

**3.3.1. Synthesis of *2,3',5',6-tetrafluoro-[1,1'-biphenyl]-4-ol***

A reaction flask was charged with 4-bromo-3,5-difluorophenol (10 g, 48 mmol) and dissolved in 250 mL of THF and 60 mL of 2M aqueous K_2_CO_3_. The resultant solution was sparged with N_2_ for 30 mins. 2, 6-difluro benzene boronic acid (8.32 g, 53 mmol) was then added to the sparged solution which was then heated to 70 ℃ (external temperature) under an atmosphere of dry nitrogen.. Once at temperature, PdXPhos (G3) (0.05 mol %) was then added in a single portion and the reaction left for 18 h with constant stirring. The reaction was then cooled, extracted with EtOAc, the aqueous and organic layers separated, the organics being dried over MgSO_4_. The suspension was filtered, concentrated *in* and purified by flash chromatography over silica gel using an isocratic run of hexane using a Combiflash NextGen300+ system to yield *3',5',6-tetrafluoro-[1,1'-biphenyl]-4-ol* as a white, crystalline solid.

*2,3',5',6-tetrafluoro-[1,1'-biphenyl]-4-ol*

Yield:	(white crystalline solid) 10.8 g, 93 %

R_F_ (DCM):	0.20

^1^H NMR (400 MHz, DMSO):	10.64 (s, 1H, Ar-O**H**), 7.26 (tt, *J* = 9.4, 2.4 Hz, 1H, Ar-**H**), 7.18 – 7.07 (m, 2H, Ar-**H**), 6.59 (ddd, *J* = 10.3, 6.6, 3.1 Hz, 2H, Ar-**H**).

^19^F NMR (376 MHz, DMSO):	-110.18 (t, *J_F-H_* = 8.6 Hz, 2F, Ar-**F**), -114.71 (d, *J_F-H_* = 10.5 Hz, 2F, Ar-**F**).

**3.3.2. Synthesis of *2,3',5',6-tetrafluoro-[1,1'-biphenyl]-4-yl 4-methylbenzenesulfonate***

*2,3',5',6-tetrafluoro-[1,1'-biphenyl]-4-ol* (9.68 g, 40 mmol) was dissolved in 100 mL of dry THF and dry NEt_3_ (12 mL, 80 mmol) under a dry nitrogen atmosphere. The flask was then cooled over an ice-water bath before TsCl (11.46 g, 60 mmol), dissolved in 50 mL of dry THF, was added dropwise to the solution over 30 minutes before the ice bath was removed and the reaction allowed to progress until complete consumption of the phenolic component as judged by TLC (circa 14 h). The reaction was then extracted with 100 mL of EtOAc and washed 3x 50 mL of 2M NaOH. The organics were combined, dried over MgSO_4_ and concentrated under reduced pressure before being purified by flash chromatography over silica gel using a gradient of hexanes:DCM using a Combiflash NextGen300+ system to yield *2,3',5',6-tetrafluoro-[1,1'-biphenyl]-4-yl 4-methylbenzenesulfonate* as a white, crystalline solid.

*2,3',5',6-tetrafluoro-[1,1'-biphenyl]-4-yl 4-methylbenzenesulfonate*

Yield:	(White crystalline solid) 15.1 g, 95 %

R_F_ (DCM):	0.84

^1^H NMR (400 MHz):	7.92 (ddd, *J* = 8.4, 2.4, 1.8 Hz, 2H, Ar-**H**), 7.41 (d, *J* = 8.5 Hz, 2H, Ar-**H**), 2.49 (s, 3H, Ar-C**H_3_**).

^9^F NMR (376 MHz):	-109.57 (t, *J_F-H_* = 7.9 Hz, 2F, Ar-**F**), -110.96 (d, *J_F-H_* = 8.3 Hz, 2F, Ar-**F**).

**3.3.3. Synthesis of *2,3',5',6-tetrafluoro-4-propyl-1,1'-biphenyl***

In a flame dried flask, *2,3',5',6-tetrafluoro-[1,1'-biphenyl]-4-yl 4-methylbenzenesulfonate* (14.65 g, 37 mmol) and Fe(acac)_3_ (1.31 g, 3.7 mmol) were dissolved in 250 mL dry THF:NMP (10:1). The solution was cooled over an ice bath before a solution of freshly prepared Grignard reagent (55 mmol) in dry Et_2_O was added dropwise to the stirred solution. The progress of the reaction was followed by ^1^H NMR with no further reaction occurring after 1.5 h. The solution was quenched with HCl (crica. 50 mL) before being extracted with EtOAc, dried over MgSO_4_ and a yellow oil was retrieved under reduced pressure. The oil was purified by flash chromatography over silica gel using a gradient of hexanes:DCM using a Combiflash NextGen300+ system, yielding 2*,3',5',6-tetrafluoro-4-propyl-1,1'-biphenyl* as a colourless oil.

*2,3',5',6-tetrafluoro-4-propyl-1,1'-biphenyl*

Yield:	(colourless oil) 7.74 g, 78 %

R_F_ (EtOAc):	0.94

^1^H NMR (400 MHz):	7.01 (m, 2H, Ar-**H**), 6.82 (ddd, *J* = 8.7, 3.1, 2.9 Hz, 2H, Ar-**H**), 2.61 (t, *J* = 7.6 Hz, 2H, Ar-C**H_2_**-CH_2_), 1.68 (h, *J* = 7.4 Hz, 2H, CH_2_-C**H_2_**-CH_3_), 0.98 (t, *J* = 7.3 Hz, 2H, CH_2_-C**H_3_**).

^19^F NMR (376 MHz):	-110.37 (t, *J_F-H_* = 8.1 Hz, 2F, Ar-**F**), -115.31 (d, *J_F-H_* = 9.2 Hz, 2F, Ar-**F**).

**3.3.4. Synthesis of *2',3,5,6'-tetrafluoro-4'-propyl-[1,1'-biphenyl]-4-carboxylic acid***

A solution of *n-butyl lithium* (1.6 M in hexanes, 25 mmol, 15 mL) was added dropwise to a stirred, cooled (-78 °C) solution of *2,3',5',6-tetrafluoro-4-propyl-1,1'-biphenyl* (4.4 g, 16.6 mmol) in anhydrous THF (150 mL) under an atmosphere of dry nitrogen. The aryl lithium solution was allowed to stir for 1.5 h at this same temperature before adding a large excess of gaseous carbon dioxide which was pre-dried by bubbling through conc. H_2_SO_4_. The solution was then allowed to slowly warm to ambient temperature (~ 2 h). The basic solution was acidified with 2M HCl (~100 ml) and extracted with EtOAc. The organics were then dried over MgSO_4_ and a white solid retrieved under reduced pressure. These were then purified by recrystallization from MeCN to give 2',3,5,6'-tetrafluoro-4'-propyl-[1,1'-biphenyl]-4-carboxylic acidacid as a fine white solid.

*2',3,5,6'-tetrafluoro-4'-propyl-[1,1'-biphenyl]-4-carboxylic acid*

Yield:	(white needles) 4.35g, 84 %

R_F_ (DCM):	0.00

^1^H NMR (400 MHz, DMSO):	8.94 (d, *J* = 8.9 Hz, 2H, Ar-**H**), 8.70 (ddd, *J* = 9.1, 3.1, 1.9 Hz, 2H, Ar-**H**), 4.19 (t, *J* = 7.3 Hz, 2H, Ar-C**H_2_**-CH_2_), 3.20 (h, *J* = 7.4 Hz, 2H, CH_2_-C**H_2_**-CH­_3_), 2.48 (t, *J* = 7.3 Hz, 3H, CH_2_-C**H_3_**).

^19^F NMR (376 MHz, DMSO):	-112.26 (d, *J_F-H_* = 9.5 Hz, 2F, Ar-**F**), -115.40 (d, *J_F-H_* = 9.6 Hz, 2F, Ar-**F**).

**3.4	General esterification procedure used in the preparation of materials FWXYZ and NCWXYZ**

The general synthetic procedure is described in **Scheme S1**. A small vial was charged with the appropriate phenol (0.5 mmol, 1.0 eq), benzoic acid (0.6 mmol, 1.1 eq.), EDC.HCl (0.75 mmol, 1.5 eqv.) and DMAP (~ 2 mol%). Dichloromethane was added (conc. ~ 0.1 M) and the suspension stirred until complete consumption of the phenol as judged by TLC. Once complete, the reaction solution was concentrated and purified by flash chromatography over silica gel with a gradient of hexane/DCM using a Combiflash NextGen300+ system. To remove ionic impurities, a pre-column containing a small amount of neutral alumina was used before samples entered the silica gel column. The chromatographed material was dissolved into the minimum quantity of DCM, filtered through a 0.2 micron PTFE filter, concentrated to dryness and finally recrystalised from either MeCN or EtOH as indicated to afford the title materials as white solids.

**F2200**

*2,3',4',5',6-pentafluoro-[1,1'-biphenyl]-4-yl 4'-(prop-1-yn-1-yl)-[1,1'-biphenyl]-4-carboxylate*

Yield:	(crystalline white solid) 183 mg, 76 %

Re-crystallisation solvent:	MeCN

^1^H NMR (400 MHz):	8.25 (ddd, *J* = 8.3, 2.1, 1.7 Hz, 2H, Ar-**H**), 7.77 (ddd, *J* = 8.2, 1.7, 1.7 Hz, 2H, Ar-**H**), 7.61 (ddd, *J* = 8.1, 2.2, 1.5 Hz, 2H, Ar-**H**), 7.34 (d, *J* = 8.0 Hz, 2H, Ar-**H**), 7.16 (t, *J* = 7.2 Hz, 2H, Ar-**H**), 7.07 – 6.98 (m, 2H, Ar-**H**), 2.69 (t, *J* = 7.8 Hz, 2H, Ar-C**H_2_**-CH_2_), 1.73 (h, *J* = 7.5 Hz, 2H, CH_2_-C**H_2_**-CH_3_), 1.02 (t, *J* = 7.3 Hz, 3H, CH2-CH_3_).

^13^C{^1^H} NMR (101 MHz):	164.24, 159.77 (dd, *J* = 250.1, 8.6 Hz), 152.33 (ddd, *J* = 250.1, 9.2, 4.5 Hz), 151.54 (t, *J* = 14.2 Hz), 147.01, 143.44, 141.04 (t, *J* = 15.9 Hz), 136.91, 130.86, 129.22, 127.18, 126.78, 124.49 – 124.20 (m), 114.83 (dd, *J* = 21.4, 6.4 Hz), 107.04 – 106.21 (m), 37.74, 24.52, 13.86.

^19^F NMR (376 MHz):	-112.54 (d, *J_F-H_* = 8.9 Hz, 2F, Ar-**F**), -134.36 (dd, *J_F-F_* = 20.5 Hz, *J_F-H_* = 8.5 Hz, 2F, Ar-**F**), -160.04 (tt, *J_F-F_* = 20.4 Hz, *J_F-H_* = 6.4 Hz, 1F, Ar-**F**).

**Fig. S9**	^1^H NMR spectra of **F2200** in CDCl_3_.

**Fig. S10**	^13^C{^1^H} NMR spectra of **F2200** in CDCl_3_.

**Fig. S11**	^19^F NMR spectra of **F2200** in CDCl_3_.

**F2010**

*3',4',5'-trifluoro-[1,1'-biphenyl]-4-yl 3-fluoro-4'-(prop-1-yn-1-yl)-[1,1'-biphenyl]-4-carboxylate*

Yield:	(crystalline white solid) 188 mg, 81 %

Re-crystallisation solvent:	MeCN

^1^H NMR (400 MHz):	8.16 (t, *J* = 7.9 Hz, 1H, Ar-**H**), 7.62 – 7.54 (m, 4H, Ar-**H**)*, 7.52 (dd, *J* = 8.2, 1.7 Hz, 1H, Ar-**H**), 7.44 (dd, *J* = 12.1, 1.7 Hz, 1H, Ar-**H**), 7.38 – 7.28 (m, 4H, Ar-**H**), 7.20 (dd, *J* = 8.4, 6.3 Hz, 2H, Ar-**H**), 2.66 (t, *J* = 7.6 Hz, 2H, Ar-C**H_2_**-CH_2_), 1.70 (h, *J* = 7.4 Hz, 2H, CH_2_-C**H_2_**-CH_3_), 0.99 (t, *J* = 7.3 Hz, 3H, CH_2_-C**H_3_**). *Overlapping Signals..

^13^C{^1^H} NMR (101 MHz):	162.75 (d, *J* = 261.5 Hz), 162.61 (d, *J* = 3.9 Hz), 151.81 (ddd, *J* = 249.8, 9.2, 3.7 Hz), 150.93, 148.81 (d, *J* = 8.8 Hz), 143.99, 136.49 (d, *J* = 4.3 Hz), 135.93 (d, *J* = 33.2 Hz), 132.98, 129.30, 128.04, 127.06, 122.45, 115.82 (t, *J* = 5.6 Hz), 115.26 (d, *J* = 23.3 Hz), 111.33 – 110.95 (m), 37.74, 24.48, 13.84.

^19^F NMR (376 MHz):	-107.57 (dd, 1F, *J_F-H_* = 12.2 Hz, *J_F-H_* = 7.5 Hz, Ar-**F**), -133.92 (dd, 2F, *J_F-F_* = 20.6 Hz, *J_F-H_* = 8.9 Hz, Ar-**F**), -162.43 (tt, 1F, *J_F-F_* = 20.3 Hz, *J_F-H_* = 6.3 Hz, Ar-**F**).

**Fig. S12**	^1^H NMR spectra of **F2010** in CDCl_3_.

**Fig. S13**	^13^C{^1^H} NMR spectra of **F2010** in CDCl_3_.

**Fig. S14**	^19^F NMR spectra of **F2010** in CDCl_3_.

**F2110**

*2,3',4',5'-tetrafluoro-[1,1'-biphenyl]-4-yl 3-fluoro-4'-propyl-[1,1'-biphenyl]-4-carboxylate*

Yield:	(white solid) 193 mg, 80 %

Re-crystallisation solvent:	MeCN

^1^H NMR (400 MHz:	8.15 (t, *J* = 7.9 Hz, 1H, Ar-**H**), 7.57 (d, *J* = 7.8 Hz, 2H, Ar-**H**), 7.54 – 7.40 (m, 3H)*, 7.31 (d, *J* = 7.8 Hz, 2H, Ar-**H**), 7.24 – 7.13 (m, 4H, Ar-**H**)*, 2.66 (t, *J* = 7.7 Hz, 2H, Ar-C**H_2_**-CH_2_), 1.69 (h, *J* = 7.5 Hz, 2H, CH_2_-C**H_2_**-CH_3_), 0.99 (t, *J* = 7.4 Hz, 3H, CH_2_-C**H_3_**). *Overlapping Signals.

^13^C{^1^H} NMR (101 MHz):	162.79 (d, *J* = 261.9 Hz), 162.18 (d, *J* = 4.3 Hz), 159.40 (d, *J* = 251.3 Hz), 151.43 (d, *J* = 11.1 Hz), 151.22 (ddd, *J* = 249.8, 9.9, 4.2 Hz), 149.08 (d, *J* = 9.2 Hz), 135.67, 132.99, 131.19 – 130.81 (m), 130.59 (d, *J* = 3.9 Hz), 129.32, 127.06, 124.13 – 123.86 (m), 122.52 (d, *J* = 3.3 Hz), 118.29 (d, *J* = 3.7 Hz), 115.30 (d, *J* = 22.7 Hz), 113.48 – 112.97 (m), 110.77 (d, *J* = 25.7 Hz), 37.74, 24.48, 13.84.

^19^F NMR (376 MHz):	-107.41 (t, 1F, *J_F-H_* = 8.5 Hz, Ar-**F**), -114.57 (t, 1F *J_F-H_* = 9.8 Hz, Ar-**F**), -134.21 (dd, 2F *J_F-F_* = 20.4 Hz, *J_F-H_* = 8.9 Hz, Ar-**F**), -161.25 (tt, 1F, *J_F-F_* = 20.5 Hz, *J_F-H_* = 6.5 Hz, Ar-**F**).

**Fig. S15**	^1^H NMR spectra of **F2110** in CDCl_3_.

**Fig. S16**	^13^C{^1^H} NMR spectra of **F2110** in CDCl_3_.

**Fig. S17**	^19^F NMR spectra of **F2110** in CDCl_3_.

**F2210**

*2,3',4',5',6-pentafluoro-[1,1'-biphenyl]-4-yl 3-fluoro-4'-propyl-[1,1'-biphenyl]-4-carboxylate*

Yield:	(white needles) 183 mg, 73 %

Re-crystallization solvent:	MeCN

^1^H NMR (400 MHz):	δ 8.13 (t, *J* = 8.2 Hz, 1H, Ar-**H**), 7.57 (ddd, *J* = 8.3, 1.9, 1.8 Hz, 2H, Ar-**H**), 7.52 (dd, *J* = 8.2, 1.7 Hz, 1H, Ar-**H**), 7.45 (dd, *J* = 12.2, 1.7 Hz, 1H, Ar-**H**), 7.32 (ddd, *J* = 8.2, 1.8, 1.8 Hz, 2H, Ar-**H**), 7.14 (t, *J* = 7.4 Hz, 2H, Ar-**H**), 7.07 – 6.97 (m, 2H, Ar-**H**), 2.67 (t, *J* = 6.7 Hz, 2H, Ar-C**H_2_**-CH_2_), 1.70 (h, *J* = 7.3 Hz, 2H, CH_2_-C**H_2_**-CH_3_), 0.99 (t, *J* = 7.3 Hz, 3H, CH_2_-C**H_3_**).

^13^C{^1^H} NMR (101 MHz):	162.81 (d, *J* = 261.9 Hz), 161.78 (d, *J* = 4.0 Hz), 159.74 (dd, *J* = 250.3, 8.6 Hz), 151.22 (t, *J* = 14.3 Hz), 151.07 (ddd, *J* = 250.0, 9.8, 3.9 Hz), 149.34 (d, *J* = 8.9 Hz), 144.18, 139.79 (dt, *J* = 253.6, 15.7 Hz), 135.56, 132.99, 129.34, 127.06, 124.32 (dd, *J* = 5.1, 3.4 Hz), 122.57 (d, *J* = 3.2 Hz), 115.32 (d, *J* = 23.0 Hz), 115.11 – 114.52 (m), 113.58 (t, *J* = 17.9 Hz), 106.99 – 106.28 (m), 37.74, 24.47, 13.83.

^19^F NMR (376 MHz):	-107.02 (dd, *J_F-H_* = 12.3 Hz, *J_F-H_* = 7.7 Hz, 1F, Ar-**F**), -112.47 (d, *J_F-H_* = 8.9 Hz, 2F, Ar-**F**), -134.34 (dd, *J_F-F_* = 20.7 Hz, *J_F-H_* = 8.5 Hz, 2F, Ar-**F**), -160.01 (tt, *J_F-F_* = 20.7 Hz, *J_F-H_* = 6.6 Hz, 1F, Ar-**F**).

**Fig. S18**	^1^H NMR spectra of **F2210** in CDCl_3_.

**Fig. S19**	^13^C{^1^H} NMR spectra of **F2210** in CDCl_3_.

**Fig. S20**	^19^F NMR spectra of **F2210** in CDCl_3_.

**F2020**

*3',4',5'-trifluoro-[1,1'-biphenyl]-4-yl 3,5-difluoro-4'-propyl-[1,1'-biphenyl]-4-carboxylate*

Yield:	(white crystalline solid) 186 mg, 77 %

Re-crystallisation solvent:	MeCN

^1^H NMR (400 MHz):	7.56 (ddd, *J* = 8.6, 3.0, 1.8 Hz, 2H, Ar-**H**), 7.53 (ddd, *J* = 8.1, 2.2, 1.3 Hz, 2H, Ar-**H**), 7.37 (ddd, *J* = 8.7, 2.6, 2.1 Hz, 2H, Ar-**H**), 7.31 (ddd, *J* = 8.0, 2.2, 1.9 Hz, 2H, Ar-**H**), 7.29 – 7.24 (m, 2H, Ar-**H**)^†^, 7.23 – 7.16 (m, 2H, Ar-**H**), 2.66 (t, *J* = 7.5 Hz, 2H, Ar-C**H_2_**-CH_2_), 1.69 (h, *J* = 7.4 Hz, 2H, CH_2_-C**H_2_**-CH_3_), 0.98 (t, *J* = 7.3 Hz, 3H, CH_2_-C**H_3_**). ^†^ Overlapping CDCl_3_.

^13^C{^1^H} NMR (101 MHz):	161.46 (dd, *J* = 258.6, 5.7 Hz), 158.74 (dd, *J* = 250.7, 8.5 Hz), 151.05 (ddd, *J* = 250.2, 10.6, 4.9 Hz), 148.26 (dt, *J* = 261.0, 15.0 Hz), 144.68, 134.76, 129.43, 126.89, 124.39 – 124.03 (m), 114.84 (dd, *J* = 16.8, 5.6 Hz), 110.52 (dd, *J* = 22.9, 2.4 Hz), 107.22 – 106.08 (m), 37.73, 24.43, 13.81.

^19^F NMR (376 MHz):	-108.55 (d, *J_F-H_* = 10.4 Hz, 2F, Ar-**F**), -133.87 (dd, *J_F-F_* = 20.4 Hz, *J_F-H_* = 8.9 Hz, 2F, Ar-**F**), -162.33 (tt, *J_F-F_* = 20.6 Hz, *J_F-H_* = 6.5 Hz, 1F, Ar-**F**).

**Fig. S21**	^1^H NMR spectra of **F2020** in CDCl_3_.

**Fig. S22**	^13^C{^1^H} NMR spectra of **F2020** in CDCl_3_.

**Fig. S23**	^19^F NMR spectra of **F2020** in CDCl_3_.

**F2220**

*2,3',4',5',6-pentafluoro-[1,1'-biphenyl]-4-yl 3,5-difluoro-4'-propyl-[1,1'-biphenyl]-4-carboxylate*

Yield:	(white needles) 173 mg, 69 %

Re-crystallisation solvent:	MeCN

^1^H NMR (400 MHz):	7.53 (d, *J* = 7.8 Hz, 2H, Ar-**H**), 7.36 – 7.23 (m, 4H, Ar-**H**)*^†^, 7.13 (t, *J* = 7.3 Hz, 2H, Ar-**H**), 7.04 (d, *J* = 8.2 Hz, 2H, Ar-**H**), 2.66 (t, *J* = 7.6 Hz, 2H, Ar-C**H_2_**-CH_2_), 1.69 (h, *J* = 7.4 Hz, 2H, CH_2_-C**H_2_**-CH_3_), 0.98 (t, *J* = 7.3 Hz, 3H, CH_2_-C**H_3_**). *Overlapping Signals, overlapping CDCl_3_.

^13^C{^1^H} NMR (101 MHz):	161.46 (dd, *J* = 258.6, 5.7 Hz), 159.03, 158.74 (dd, *J* = 250.7, 8.5 Hz), 151.05 (ddd, *J* = 250.2, 10.6, 4.9 Hz), 148.26 (dt, *J* = 261.0, 15.0 Hz), 144.68, 134.76, 129.43, 126.89, 124.39 – 124.03 (m), 114.84 (dd, *J* = 16.8, 5.6 Hz), 110.52 (dd, *J* = 22.9, 2.4 Hz), 107.22 – 106.08 (m), 37.73, 24.43, 13.81.

^19^F NMR (376 MHz):	-107.97 (d, *J_F-H_* = 10.8 Hz, 2F, Ar-**F**), -112.20 (d, *J_F-H_* = 8.8 Hz, 2F, Ar-**F**), -134.30 (dd, *J_F-F_* = 20.6 Hz, *J_F-H_* = 8.7 Hz, 2F, Ar-**F**), -159.92 (tt, *J_F-F_* = 20.5 Hz, *J_F-H_* = 6.5 Hz, 1F, Ar-**F**).

**Fig. S24**	^1^H NMR spectra of **F2220** in CDCl_3_.

**Fig. S25**	^13^C{^1^H} NMR spectra of **F2220** in CDCl_3_.

**Fig. S26**	^19^F NMR spectra of **F2220** in CDCl_3_.

**F2021**

*3',4',5'-trifluoro-[1,1'-biphenyl]-4-yl 2',3,5-trifluoro-4'-propyl-[1,1'-biphenyl]-4-carboxylate*

Yield:	(white crystalline solid) 155 mg, 31 %

Re-crystallisation solvent:	MeCN

^1^H NMR (400 MHz:	7.56 (ddd, *J* = 8.5, 3.0, 1.8 Hz, 2H, Ar-**H**), 7.41 – 7.33 (m, 3H, Ar-**H**)*, 7.30 – 7.23 (m, 2H, Ar-**H**)^†^, 7.23 – 7.15 (m, 2H, Ar-**H**)*, 7.09 (dd, *J* = 8.0, 1.6 Hz, 1H, Ar-**H**), 7.03 (dd, *J* = 12.0, 1.6 Hz, 1H, Ar-**H**), 2.65 (t, *J* = 7.6 Hz, 2H, Ar-C**H_2_**-CH_2_), 1.69 (h, *J* = 7.3 Hz, 2H, CH_2_-C**H_2_**-CH_3_), 0.99 (t, *J* = 7.3 Hz, 3H, CH_2_-C**H_3_**). ^†^ Overlapping CDCl_3,_ *Overlapping Signals.

^13^C{^1^H} NMR (101 MHz):	162.42 – 159.57 (m)*, 159.65 (d, *J* = 250.2 Hz), 151.68 (ddd, *J* = 249.9, 9.9, 4.5 Hz), 150.61, 146.93 (d, *J* = 7.8 Hz), 142.28 (t, *J* = 10.8 Hz), 139.39 (dt, *J* = 252.2, 15.1 Hz), 136.40, 129.77 (d, *J* = 3.0 Hz), 128.10, 125.01 (d, *J* = 3.0 Hz), 122.85 (dt, *J* = 12.8, 2.1 Hz) 122.33, 116.55, 116.33, 112.63 (dt, *J* = 22.5, 3.3 Hz), 111.36 – 110.97 (m), 108.46 (t, *J* = 17.1 Hz), 37.54, 24.14, 13.72. *Overlapping Signals.

^19^F NMR (376 MHz):	-109.08 (d, 2F, *J_F-H_* = 10.4 Hz, Ar-**F**), -117.58 (t,1F *J_F-H_* = 9.1 Hz , Ar-**F**), -133.86 (dd, 2F *J_F-F_* = 20.6 Hz, *J_F-H_* = 8.7 Hz, Ar-**F**), -162.32 (tt, 1F, *J_F-F_* = 14.1 Hz, *J_F-H_* 6.6 Hz, Ar-**F**).

**Fig. S27**	^1^H NMR spectra of **F2021** in CDCl_3_.

**Fig. S28**	^13^C{^1^H} NMR spectra of **F2021** in CDCl_3_.

**Fig. S29**	^19^F NMR spectra of **F2021** in CDCl_3_.

**F2121**

*2,3',4',5'-tetrafluoro-[1,1'-biphenyl]-4-yl 2',3,5-trifluoro-4'-propyl-[1,1'-biphenyl]-4-carboxylate*

Yield:	(white crystalline solid) 161 mg, 62 %

Re-crystallisation solvent:	MeCN

^1^H NMR (400 MHz):	7.45 (t, *J* = 8.6 Hz, 1H, Ar-**H**), 7.37 (t, *J* = 8.0 Hz, 1H, Ar-**H**), 7.29 – 7.23 (m, 2H, Ar-**H**)^†^, 7.23 – 7.14 (m, 4H, Ar-**H**)*, 7.09 (dd, *J* = 7.9, 1.7 Hz, 1H, Ar-**H**), 7.03 (dd, *J* = 11.9, 1.6 Hz, 1H, Ar-**H**), 2.65 (t, *J* = 7.4 Hz, 2H, Ar-C**H_2_**-CH_2_), 1.69 (h, *J* = 7.4 Hz, 2H, CH_2_-C**H_2_**-CH_3_), 0.98 (t, *J* = 7.3 Hz, 3H, CH_2_-C**H_3_**). ^†^ Overlapping CDCl_3_ peak; *Overlapping Signals.

^13^C{^1^H} NMR (101 MHz):	162.40 (dd, *J* = 258.3, 6.8 Hz), 160.27 (dd, *J* = 250.8, 19.6 Hz), 159.33, 152.47 (dd, *J* = 9.9, 4.1 Hz), 151.03 (d, *J* = 11.0 Hz), 149.92 (t, *J* = 4.0 Hz), 147.02 (d, *J* = 7.7 Hz), 142.59 (t, *J* = 11.4 Hz), , 130.67 (d, *J* = 3.7 Hz), 129.76 (d, *J* = 3.0 Hz), 125.04 (d, *J* = 3.2 Hz), 124.31 (d, *J* = 13.9 Hz), 122.71 (d, *J* = 11.9 Hz), 118.16 (d, *J* = 3.7 Hz), 116.57, 116.35, 113.42 – 113.01 (m), 112.69 (tt, *J* = 23.5, 3.6 Hz), 110.79, 110.54, 108.01 (t, *J* = 16.8 Hz), 37.55, 24.13, 13.72.

^19^F NMR (376 MHz):	-108.80 (d, 2F, *J_F-H_* = 10.3 Hz, Ar-**F**), -114.32 (t, 1F, *J_F-H_* = 9.7 Hz, Ar-**F**), -117.56 (dd, 1F, *J_F-H_* = 11.8 Hz, *J_F-H_* = 8.1 Hz, Ar-**F**), -134.15 (dd, 2F, *J_F-F_* = 20.7 Hz, *J_F-H_* = 8.7 Hz, Ar-**F**), -161.14 (tt, 1F, *J_F-F_* = 20.6 Hz, *J_F-H_* = 6.5 Hz, Ar-**F**).

**Fig. S30**	^1^H NMR spectra of **F2121** in CDCl_3_.

**Fig. S31**	^13^C{^1^H} NMR spectra of **F2121** in CDCl_3_.

**Fig. S32**	^19^F NMR spectra of **F2121** in CDCl_3_.

**F2221**

*2,3',4',5',6-pentafluoro-[1,1'-biphenyl]-4-yl 2',3,5-trifluoro-4'-propyl-[1,1'-biphenyl]-4-carboxylate*

Yield:	(white needles) 145 mg, 54 %

Re-crystallisation solvent:	MeCN

^1^H NMR (400 MHz):	7.37 (t, *J* = 8.0 Hz, 1H, Ar-**H**), 7.31 – 7.24 (m, 2H, Ar-**H**)^†^, 7.18 – 6.99 (m, 6H, Ar-**H**)*, 2.65 (t, *J* = 7.7 Hz, 2H, Ar-C**H_2_**-CH_2_), 1.69 (h, *J* = 7.5 Hz, 2H, CH_2_-C**H_2_**-CH_3_), 0.98 (t, *J* = 7.3 Hz, 3H, CH_2_-C**H_3_**). ^†^ Overlapping CDCl_3_ peak; *Overlapping Signals.

^13^C{^1^H} NMR (101 MHz):	162.46 (dd, *J* = 258.8, 5.8 Hz), 160.31 (dd, *J* = 250.5, 8.8 Hz), 159.59 (d, *J* = 250.5 Hz), 158.92, 151.10 (ddd, *J* = 243.9, 9.7, 3.6 Hz), 150.77 (t, *J* = 14.3 Hz), 147.13 (d, *J* = 8.0 Hz), 142.92 (d, *J* = 9.9 Hz), 138.56 (dd, *J* = 254.5, 16.4 Hz), 129.74 (d, *J* = 3.1 Hz), 125.06 (d, *J* = 3.1 Hz), 124.18 (d, *J* = 8.9 Hz), 122.75 (d, *J* = 12.5 Hz), 116.59, 116.37, 115.01 – 114.64 (m_apparent_), 112.79 (dt, *J* = 23.2, 3.4 Hz), 107.57 (t, *J* = 17.6 Hz), 106.86 – 106.31 (m_apparent_), 37.55, 24.13, 13.71.

^19^F NMR (376 MHz):	-108.52 (d, 2F, *J_F-H_* = 10.5 Hz, Ar-**F**), -112.15 (d, 2F *J_F-H_* = 8.8 Hz, Ar-**F**), -117.52 (dd, 1F *J_F-H_* = 11.9 Hz, *J_F-H_* 8.1 Hz, Ar-**F**), -134.29 (dd, 2F *J_F-F_* = 20.6 Hz, *J_F-H_* = 8.4 Hz, Ar-**F**), -159.91 (tt, 1F, *J_F-H_* = 20.6, *J_F-H_* = 6.6 Hz, Ar-**F**).

**Fig. S33**	^1^H NMR spectra of **F2221** in CDCl_3_**.**

**Fig. S34**	^13^C{^1^H} NMR spectra of **F2221** in CDCl_3_**.**

**Fig. S35**	^19^F NMR spectra of **F2221** in CDCl_3_**.**

**F2022**

*3',4',5'-trifluoro-[1,1'-biphenyl]-4-yl 2',3,5,6'-tetrafluoro-4'-propyl-[1,1'-biphenyl]-4-carboxylate*

Yield:	(white crystalline solid) 130 mg, 50 %

Re-crystallisation solvent:	MeCN

^1^H NMR (400 MHz):	7.57 (ddd, *J* = 8.7, 2.8, 2.1 Hz, 2H, Ar-**H**), 7.37 (ddd, *J* = 8.8, 2.7, 2.2 Hz, 2H, Ar-**H**), 7.23 – 7.15 (m, 4H, Ar-**H**)*, 6.87 (ddd, *J* = 9.0, 2.8, 2.2 Hz, 2H, Ar-**H**), 2.63 (t, *J* = 7.4 Hz, 2H, Ar-C**H_2_**-CH_2_), 1.69 (h, *J* = 7.3 Hz, 2H, CH_2_-C**H_2_**-CH_3_), 0.99 (t, *J* = 7.3 Hz, 3H, CH_2_-C**H_3_**). *Overlapping signals.

^13^C{^1^H} NMR (101 MHz):	160.54 (dd, *J* = 257.1, 7.0 Hz), 159.43, 158.23 (dd, *J* = 250.7, 6.4 Hz), 150.93 (ddd, *J* = 249.8, 10.1, 4.5 Hz), 147.15 (t, *J* = 9.6 Hz), 138.93 (dt, *J* = 252.6, 15.6 Hz), 136.63 – 136.22 (m), 135.85 (t, *J* = 11.2 Hz), 128.13, 122.30, 114.53, 114.43 (dd, *J* = 23.8, 2.6 Hz), 112.51 (t, *J* = 17.8 Hz), 111.97 (dd, *J* = 19.1, 6.0 Hz), 111.16 (dd, *J* = 15.7, 5.9 Hz), 109.27 (t, *J* = 17.2 Hz), 37.68, 23.86, 13.63.

^19^F NMR (376 MHz):	-109.54 (d, *J_F-H_* = 10.0 Hz, 2F, Ar-**F**), -114.95 (d, *J_F-H_* = 9.5 Hz, 2F, Ar-**F**), -133.86 (dd, *J_F-F_* = 20.4 Hz, *J_F-H_* = 8.8 Hz, 2F, Ar-**F**), -162.32 (tt, *J_F-F_* = 20.4 Hz, *J_F-H_* = 6.5 Hz, 1F, Ar-**F**).

**Fig. S36**	^1^H NMR spectra of **F2022** in CDCl_3_**.**

**Fig. S37**	^13^C{^1^H} NMR spectra of **F2022** in CDCl_3_**.**

**Fig. S38**	^19^F NMR spectra of **F2022** in CDCl_3_**.**

**F2122**

*2,3',4',5'-tetrafluoro-[1,1'-biphenyl]-4-yl 2',3,5,6'-tetrafluoro-4'-propyl-[1,1'-biphenyl]-4-carboxylate*

Yield:	(white solid) 140 mg, 52 %

Re-crystallisation solvent:	MeCN

^1^H NMR (400 MHz):	7.44 (t, *J* = 8.3 Hz, 1H, Ar-**H**), 7.23 – 7.15 (m, 6H, Ar-**H**)*, 6.87 (ddd, *J* = 9.1, 5.3, 2.1 Hz, 2H, Ar-**H**), 2.64 (t, *J* = 7.5 Hz, 2H, Ar-C**H_2_**-CH_2_), 1.69 (h, *J* = 7.6 Hz, 2H, CH_2_-C**H_2_**-CH_3_), 0.99 (t, *J* = 7.3 Hz, 3H, CH_2_-C**H_3_**). *Overlapping Signals.

^13^C{^1^H} NMR (101 MHz):	160.74 (dd, *J* = 258.1, 6.5 Hz), 159.22, 158.74 (d, *J* = 250.3 Hz), 158.14 (dd, *J* = 250.9, 3.3 Hz), 151.23 (ddd, *J* = 250.0, 9.9, 4.1 Hz), 150.99 (d, *J* = 11.0 Hz), 147.25 (t, *J* = 9.5 Hz), 139.56 (dt, *J* = 252.8, 15.5 Hz), 136.18 (t, *J* = 11.5 Hz), 130.70 (d, *J* = 3.8 Hz), 124.61 – 124.17 (m), 118.14 (d, *J* = 3.8 Hz), 114.45 (dd, *J* = 23.6, 2.3 Hz), 113.42 – 113.06 (m), 112.43 (t, *J* = 10.8 Hz), 111.99 (dd, *J* = 19.1, 6.1 Hz), 110.65 (d, *J* = 26.1 Hz), 108.81 (t, *J* = 16.8 Hz), 37.68, 23.85, 13.63.

^19^F NMR (376 MHz):	-109.27 (d, *J_F-H_* = 10.0 Hz, 2F, Ar-**F**), -114.26 (t, *J_F-H_* = 9.4 Hz, 1F, Ar-**F**), -114.94 (d, *J_F-H_* = 9.6 Hz, 2F, Ar-**F**), -134.14 (dd, *J_F-F_* = 20.6 Hz, *J_F-H_* = 8.7 Hz, 2F, Ar-**F**), -161.13 (tt, *J_F-F_* = 20.6 Hz, *J_F-H_* = 6.5 Hz, 1F, Ar-**F**).

**Fig. S39**	^1^H NMR spectra of **F2122** in CDCl_3_**.**

**Fig. S40**	^13^C{^1^H} NMR spectra of **F2122** in CDCl_3_**.**

**Fig. S41**	^19^F NMR spectra of **F2122** in CDCl_3_**.**

**F2222**

*2,3',4',5',6-pentafluoro-[1,1'-biphenyl]-4-yl 2',3,5,6'-tetrafluoro-4'-propyl-[1,1'-biphenyl]-4-carboxylate*

Yield:	(white needles) 131 mg, 47 %

Re-crystallisation solvent:	MeCN

^1^H NMR (400 MHz):	7.20 (d, *J* = 9.1 Hz, 2H, Ar-**H**), 7.13 (t, *J* = 7.2 Hz, 2H, Ar-**H**), 7.03 (ddd, *J* = 8.0, 3.3, 3.2 Hz, 2H, Ar-**H**), 6.87 (ddd, *J* = 9.1, 4.8, 2.4 Hz, 2H, Ar-**H**), 2.64 (t, *J* = 7.4 Hz, 2H, Ar-C**H_2_**-CH_2_), 1.69 (h, *J* = 7.4 Hz, 2H, CH_2_-C**H_2_**-CH_3_), 0.99 (t, *J* = 7.3 Hz, 3H, CH_2_-C**H_3_**).

^13^C{^1^H} NMR (101 MHz):	161.21 (dd, *J* = 139.3, 6.1 Hz), 158.84 (dd, *J* = 250.7, 8.3 Hz), 158.80, 158.24 (dd, *J* = 250.6, 6.1 Hz), 151.13 (ddd, *J* = 249.3, 8.8, 3.9 Hz), 150.73 (t, *J* = 14.4 Hz), 147.34 (t, *J* = 9.5 Hz), 139.83 (dt, *J* = 253.9, 15.1 Hz), 136.51 (t, *J* = 11.2 Hz), 124.17 (m), 114.67 (m), 113.95 (d, *J* = 16.8 Hz), 112.37 (t, *J* = 12.7 Hz), 112.01 (dd, *J* = 24.3, 5.5 Hz), 108.38 (t, *J* = 16.5 Hz), 106.54 (dd, *J* = 28.3, 8.7 Hz), 37.68, 23.84, 13.63.

^19^F NMR (376 MHz):	-109.00 (d, *J_F-H_* = 10.1 Hz, 2F, Ar-**F**), -112.10 (d, *J_F-H_* = 8.8 Hz, 2F, Ar-**F**), -114.94 (d, *J_F-H_* = 9.8 Hz, 2F, Ar-**F**), -134.29 (dd, *J_F-F_* = 20.7 Hz, *J_F-H_* = 8.4 Hz, 2F, Ar-**F**), -159.90 (tt, *J_F-F_* = 20.7 Hz, *J_F-H_* = 6.5 Hz, 1F, Ar-**F**).

**Fig. S42**	^1^H NMR spectra of **F2222** in CDCl_3_**.**

**Fig. S43**	^13^C{^1^H} NMR spectra of **F2222** in CDCl_3_**.**

**Fig. S44**	^19^F NMR spectra of **F2222** in CDCl_3_**.**

**NC2100**

*4'-cyano-2,3',5'-trifluoro-[1,1'-biphenyl]-4-yl 4'-propyl-[1,1'-biphenyl]-4-carboxylate*

Yield:	(white crystalline solid) 193 mg, 82 %

Re-crystallisation solvent:	MeCN

^1^H NMR (400 MHz):	8.27 (d, *J* = 8.0 Hz, 2H, Ar-**H**), 7.78 (d, *J* = 8.1 Hz, 2H, Ar-**H**), 7.62 (d, *J* = 7.8 Hz, 2H, Ar-**H**), 7.52 (t, *J* = 8.6 Hz, 1H, Ar-**H**), 7.38 – 7.18 (m, 6H, Ar-**H**)^*†^, 2.69 (t, *J* = 7.6 Hz, 2H, Ar-C**H_2_**-CH_2_), 1.73 (h, *J* = 7.4 Hz, 2H, CH_2_-C**H_2_**-CH_3_), 1.02 (t, *J* = 7.3 Hz, 3H, CH_2_-C**H_3_**). ^*^Overlapping signals, ^†^Overlapping CDCl_3_.

^13^C{^1^H} NMR (126 MHz):	164.47, 163.08 (dd, *J* = 260.9, 5.2 Hz), 159.56 (d, *J* = 252.8 Hz), 152.99 (d, *J* = 11.2 Hz), 146.92, 143.44, 143.16 (t, *J* = 10.2 Hz), 136.92, 130.85, 130.55 (d, *J* = 3.6 Hz), 129.22, 127.17 (d, *J* = 2.8 Hz), 126.96, 122.83 (dt, *J* = 12.3, 2.2 Hz), 118.76 (d, *J* = 3.6 Hz), 112.61 (dt, *J* = 20.7, 3.7 Hz), 111.14 (d, *J* = 25.7 Hz), 109.17, 91.42 (t, *J* = 19.3 Hz), 37.75, 24.53, 13.87.

^19^F NMR (376 MHz):	-103.53 (d, *J_F-H_* = 9.4 Hz, 2F, Ar-**F**), -113.45 (t, *J_F-H_* = 9.8 Hz, 1F, Ar-**F**).

**Fig. S45**	^1^H NMR spectra of **NC2100** in CDCl_3_**.**

**Fig. S46**	^13^C{^1^H} NMR spectra of **NC2100** in CDCl_3_**.**

**Fig. S47**	^19^F NMR spectra of **NC2100** in CDCl_3_**.**

**NC1200**

*4'-cyano-2,3',6-trifluoro-[1,1'-biphenyl]-4-yl 4'-propyl-[1,1'-biphenyl]-4-carboxylate*

Yield:	(white crystalline solid) 205 mg, 87 %

Re-crystallisation solvent:	MeCN

^1^H NMR (501 MHz):	8.24 (ddd, *J* = 8.3, 2.1, 1.7 Hz, 2H, Ar-**H**), 7.76 (dt, *J* = 8.3, 1.8 Hz, 2H, Ar-**H**), 7.73 (t, *J* = 6.9 Hz, 1H, Ar-**H**), 7.59 (ddd, *J* = 8.0, 2.0, 1.9 Hz, 2H, Ar-**H**), 7.44 – 7.36 (m, 2H, Ar-**H**), 7.31 (ddd, *J* = 8.1, 2.1, 2.1 Hz, 2H, Ar-**H**), 7.03 (ddd, *J* = 8.2, 3.2, 2.7 Hz, 2H, Ar-**H**), 2.66 (t, *J* = 6.8 Hz, 2H, Ar-C**H_2_**-CH_2_), 1.70 (h, *J* = 7.3 Hz, 2H, CH_2_-C**H_2_**-CH_3_), 0.99 (t, *J* = 7.3 Hz, 3H, CH_2_-C**H_3_**).

^13^C{^1^H} NMR (126 MHz):	164.18, 162.83 (d, *J* = 259.2 Hz), 159.72 (dd, *J* = 251.2, 8.7 Hz), 152.24 (t, *J* = 14.3 Hz), 147.09, 143.49, 136.86136.05 (d, *J* = 8.7 Hz), 133.25, 130.89, 129.23, 127.19 (d, *J* = 1.5 Hz), 126.99 – 126.79 (m), 118.52 (dt, *J* = 20.8, 2.0 Hz), 113.40 (td, *J* = 18.0, 2.0 Hz), 107.14 – 106.49 (m), 101.25 (d, *J* = 15.5 Hz), 37.75, 24.53, 13.87.

^19^F NMR (376 MHz):	-106.13 (t, *J_F-H_* = 8.3 Hz, 1F, Ar-**F**), -112.22 (d, *J_F-H_* = 9.0 Hz, 2F, Ar-**F**).

**Fig. S48**	^1^H NMR spectra of **NC1200** in CDCl_3_**.**

**Fig. S49**	^13^C{^1^H} NMR spectra of **NC1200** in CDCl_3_**.**

**Fig. S50**	^19^F NMR spectra of **NC1200** in CDCl_3_**.**

**NC2200**

*4'-cyano-2,3',5',6-tetrafluoro-[1,1'-biphenyl]-4-yl 4'-propyl-[1,1'-biphenyl]-4-carboxylate*

Yield:	(white solid) 196 mg, 80 %

Re-crystallisation solvent:	MeCN

^1^H NMR (400 MHz):	8.24 (ddd, *J* = 8.4, 1.9, 1.8 Hz, 2H, Ar-**H**), 7.76 (ddd, *J* = 8.3, 1.6, 1.6 Hz, 2H, Ar-**H**), 7.59 (ddd, *J* = 8.2, 1.8, 1.5 Hz, 2H, Ar-**H**), 7.32 (ddd, *J* = 8.1, 1.9, 1.8 Hz, 2H, Ar-**H**), 7.23 (d, *J* = 8.3 Hz, 2H, Ar-**H**), 7.05 (ddd, *J* = 8.3, 1.4, 1.3 Hz, 2H, Ar-**H**), 2.66 (t, *J* = 7.4 Hz, 2H, Ar-C**H_2_**-CH_2_), 1.70 (h, *J* = 7.4 Hz, 2H, CH_2_-C**H_2_**-CH_3_), 0.99 (t, *J* = 7.3 Hz, 3H, CH_2_-C**H_3_**).

^13^C{^1^H} NMR (101 MHz):	164.18, 162.85 (dd, *J* = 261.3, 5.2 Hz), 159.62 (dd, *J* = 251.8, 8.3 Hz), 152.77 (t, *J* = 14.0 Hz), 147.15, 143.51, 136.87, 136.83 (t, *J* = 10.9 Hz), 130.90, 129.23, 127.21, 127.18, 126.56, 114.28 (dd, *J* = 21.0, 2.6 Hz), 112.58 (t, *J* = 17.9 Hz), 107.32 – 106.74 (m), 92.19 (t, *J* = 19.1 Hz), 37.74, 24.52, 13.86.

^19^F NMR (376 MHz):	-103.69 (d, *J_F-H_* = 8.8 Hz, 2F, Ar-**F**), -111.90 (d, *J_F-H_* = 9.5 Hz, 2F, Ar-**F**).

**Fig. S51**	^1^H NMR spectra of **NC2200** in CDCl_3_**.**

**Fig. S52**	^13^C{^1^H} NMR spectra of **NC2200** in CDCl_3_**.**

**Fig. S53**	^19^F NMR spectra of **NC2200** in CDCl_3_**.**

**NC2110**

*4'-cyano-2,3',5'-trifluoro-[1,1'-biphenyl]-4-yl 3-fluoro-4'-propyl-[1,1'-biphenyl]-4-carboxylate*

Yield:	(white crystalline solid) 193 mg, 79 %

Re-crystallisation solvent:	MeCN

^1^H NMR (400 MHz):	8.08 (t, *J* = 7.9 Hz, 1H, Ar-**H**), 7.50 (d, *J* = 8.1 Hz, 2H, Ar-**H**), 7.48 – 7.34 (m, 2H, Ar-**H**)*, 7.30 – 7.10 (m, 6H, Ar-**H**)*^†^, 2.60 (t, *J* = 7.6 Hz, 2H, Ar-C**H_2_**-CH_2_), 1.63 (h, *J* = 7.4 Hz, 2H, CH_2_-C**H_2_**-CH_3_), 0.92 (t, *J* = 7.3 Hz, 3H, CH_2_-CH_3_). * Overlapping Signals, ^†^Overlapping CDCl_3_.

^13^C{^1^H} NMR (101 MHz):	164.36, 164.11, 164.23 (dd, *J* = 260.6, 5.4 Hz), 162.04 (d, *J* = 4.1 Hz), 161.70 (d, *J* = 261.6 Hz), 159.53 (d, *J* = 252.7 Hz), 152.59 (d, *J* = 11.4 Hz), 149.25 (d, *J* = 9.1 Hz), 144.16, 143.11 (t, *J* = 9.9 Hz), 135.59, 133.00, 130.54 (d, *J* = 3.7 Hz), 129.34, 127.06, 122.92 (d, *J* = 12.5 Hz), 122.56 (d, *J* = 3.2 Hz), 118.71 (d, *J* = 3.7 Hz), 115.32 (d, *J* = 23.2 Hz), 112.62 (dt, *J* = 21.1, 3.4 Hz), 111.12 (d, *J* = 25.7 Hz), 91.46 (t, *J* = 19.6 Hz), 37.74, 24.47, 13.84.

^19^F NMR (376 MHz):	-103.50 (d, *J_F-H_* = 9.4 Hz, 2F, Ar-**F**), -107.17 (dd, *J_F-H_* = 12.3 Hz, *J_F-H_* = 7.6 Hz, 1F, Ar-**F**), -113.40 (t, *J_F-H_* = 9.8 Hz, 1F, Ar-**F**).

**Fig. S54**	^1^H NMR spectra of **NC2110** in CDCl_3_**.**

**Fig. S55**	^13^C{^1^H} NMR spectra of **NC2110** in CDCl_3_**.**

**Fig. S56**	^19^F NMR spectra of **NC2110** in CDCl_3_**.**

**NC1210**

*4'-cyano-2,3',6-trifluoro-[1,1'-biphenyl]-4-yl 3-fluoro-4'-propyl-[1,1'-biphenyl]-4-carboxylate*

Yield:	(white solid) 183 mg, 75 %

Re-crystallisation solvent:	MeCN

^1^H NMR (400 MHz):	8.14 (t, *J* = 7.8 Hz, 1H, Ar-**H**), 7.73 (t, *J* = 7.3 Hz, 1Hm Ar-**H**), 7.62 – 7.35 (m, 6H, Ar-**H**)*, 7.32 (d, *J* = 7.8 Hz, 2H, Ar-**H**), 7.05 (d, *J* = 8.4 Hz, 2H, Ar-**H**), 2.66 (t, *J* = 7.6 Hz, 2H, Ar-C**H_2_**-CH_2_), 1.70 (h, *J* = 7.4 Hz, 2H, CH_2_-C**H_2_**-CH_3_), 0.99 (t, *J* = 7.3 Hz, 3H, CH_2_-C**H_3_**). * Overlapping Signals.

^13^C{^1^H} NMR (101 MHz):	162.83 (d, *J* = 260.8 Hz), 161.70 (d, *J* = 4.5 Hz), 159.69 (dd, *J* = 251.2, 8.6 Hz), 151.83 (t, *J* = 14.3 Hz), 149.43 (d, *J* = 9.0 Hz), 144.22, 136.04, 136.00 (d, *J* = 8.7 Hz), 133.25, 133.00, 129.35, 127.07, 126.98 (d, *J* = 18.5 Hz)*, 122.60 (d, *J* = 3.3 Hz), 118.51 (d, *J* = 20.7 Hz), 115.35 (d, *J* = 23.0 Hz), 114.85 (d, *J* = 9.3 Hz), 113.73, 107.19 – 106.36 (m), 101.28 (d, *J* = 15.5 Hz), 37.74, 24.47, 13.83. * Overlapping Signals.

^19^F NMR (376 MHz):	-106.12 (t, *J_F-H_* = 8.3 Hz, 1F, Ar-**F**), -106.96 (dd, *J_F-H_* = 12.2 Hz, *J_F-H_* = 7.6 Hz, 1F. Ar-**F**), -112.14 (d, *J_F-H_* = 9.3 Hz, 2F, Ar-**F**).

**Fig. S57**	^1^H NMR spectra of **NC2110** in CDCl_3_**.**

**Fig. S58**	^13^C{^1^H} NMR spectra of **NC2110** in CDCl_3_**.**

**Fig. S59**	^19^F NMR spectra of **NC2110** in CDCl_3_**.**

**NC2210**

*4'-cyano-2,3',5',6-tetrafluoro-[1,1'-biphenyl]-4-yl 3-fluoro-4'-propyl-[1,1'-biphenyl]-4-carboxylate*

Yield:	(white solid) 190 mg, 75 %

Re-crystallisation solvent:	MeCN

^1^H NMR (400 MHz):	8.13 (t, *J* = 7.9 Hz, 1H, Ar-**H**), 7.57 (d, *J* = 8.1 Hz, 2H, Ar-**H**), 7.53 (dd, *J* = 8.3, 1.7 Hz, 1H, Ar-**H**), 7.45 (dd, *J* = 12.1, 1.7 Hz, 1H, Ar-**H**), 7.32 (d, *J* = 7.9 Hz, 2H, Ar-**H**), 7.23 (d, *J* = 8.8 Hz, 2H, Ar-**H**), 7.07 (ddd, *J* = 8.4, 5.6, 3.0 Hz, 2H, Ar-**H**), 2.66 (t, *J* = 7.6 Hz, 2H, Ar-C**H_2_**-CH_2_), 1.70 (h, *J* = 7.4 Hz, 2H, CH_2_-C**H_2_**-CH_3_), 0.98 (t, *J* = 7.3 Hz, 3H, CH_2_-C**H_3_**).

^13^C{^1^H} NMR (101 MHz):	162.84 (dd, *J* = 261.6, 5.0 Hz), 161.59 (d, *J* = 3.9 Hz), 159.60 (dd, *J* = 252.0, 8.2 Hz), 152.20 (t, *J* = 14.6 Hz), 149.51 (d, *J* = 8.9 Hz), 144.25, 136.72 (t, *J* = 10.6 Hz), 135.52, 133.01, 129.35, 127.07, 122.62 (d, *J* = 3.3 Hz), 115.36 (d, *J* = 22.9 Hz), 114.75 (d, *J* = 9.6 Hz), 114.28 (d, *J* = 21.1 Hz), 112.71 (t, *J* = 18.8 Hz), 108.98, 107.28 – 106.69 (m), 92.22 (t, *J* = 18.7 Hz), 37.73, 24.47, 13.83.

^19^F NMR (376 MHz):	-103.67 (d, *J_F-H_* = 9.2 Hz, 2F, Ar-**F**), -106.90 (dd, *J_F-H_* = 12.2 Hz, *J_F-H_* = 7.6 Hz, 1F), -111.82 (d, *J_F-H_* = 9.5 Hz, 2F, Ar-**F**).

**Fig. S60**	^1^H NMR spectra of **NC2210** in CDCl_3_**.**

**Fig. S61**	^13^C{^1^H} NMR spectra of **NC2210** in CDCl_3_**.**

**Fig. S62**	^19^F NMR spectra of **NC2210** in CDCl_3_**.**

**NC2120**

*4'-cyano-2,3',5'-trifluoro-[1,1'-biphenyl]-4-yl 3,5-difluoro-4'-propyl-[1,1'-biphenyl]-4-carboxylate*

Yield:	(white solid) 203 mg, 80 %

Re-crystallisation solvent:	MeCN

^1^H NMR (400 MHz):	7.56 – 7.47 (m, 3H, Ar-**H**)*, 7.34 – 7.22 (m, 8H, Ar-**H**)*^†^, 2.66 (t, *J* = 7.4 Hz, 2H, Ar-C**H_2_**-CH_2_), 1.69 (h, *J* = 7.3 Hz, 2H, CH_2_-C**H_2_**-CH_3_), 0.98 (t, *J* = 7.3 Hz, 3H, CH_2_-C**H_3_**). *Overlapping Signals, ^†^ Overlapping CDCl_3_.

^13^C{^1^H} NMR (101 MHz):	164.39 (dd, *J* = 260.5, 5.3 Hz), 162.35 (dd, *J* = 258.3, 5.8 Hz), 159.26 (d, *J* = 252.3 Hz), 152.20 (d, *J* = 11.4 Hz), 148.15 (t, *J* = 10.4 Hz), 144.66, 143.07 (t, *J* = 10.2 Hz), 134.77, 130.61 (d, *J* = 3.5 Hz), 129.43, 126.89, 123.26 (d, *J* = 12.7 Hz), 118.61 (d, *J* = 3.7 Hz), 112.68 (dt, *J* = 20.6, 3.4 Hz), 111.04 (d, *J* = 25.8 Hz), 110.50 (dd, *J* = 23.4, 3.0 Hz), 109.11, 107.12 (t, *J* = 16.9 Hz), 91.53 (t, *J* = 19.3 Hz), 37.72, 24.43, 13.81.

^19^F NMR (376 MHz):	-103.45 (d, *J_F-H_* = 9.1 Hz, 2F, Ar-**F**), -108.08 (d, *J_F-H_* = 10.7 Hz, 2F, Ar-**F**), -113.21 (t, *J_F-H_* = 9.7 Hz, 1F, Ar-**F**).

**Fig. S63**	^1^H NMR spectra of **NC2120** in CDCl_3_**.**

**Fig. S64**	^13^C{^1^H} NMR spectra of **NC2120** in CDCl_3_**.**

**Fig. S65**	^19^F NMR spectra of **NC2120** in CDCl_3_**.**

**NC1220**

*4'-cyano-2,3',6-trifluoro-[1,1'-biphenyl]-4-yl 3,5-difluoro-4'-propyl-[1,1'-biphenyl]-4-carboxylate*

Yield:	(white needles) 175 mg, %

Re-crystallisation solvent:	MeCN

^1^H NMR (400 MHz):	7.73 (t, *J* = 7.3 Hz, 1H, Ar-**H**), 7.52 (d, *J* = 8.0 Hz, 2H, Ar-**H**), 7.39 (m, 2H, Ar-**H**), 7.34 – 7.27 (m, 4H, Ar-**H**)*, 7.07 (ddd, *J* = 8.5, 5.9, 3.5 Hz, 2H, Ar-**H**), 2.66 (t, *J* = 7.6 Hz, 2H, Ar-C**H_2_**-CH_2_), 1.69 (h, *J* = 7.4 Hz, 2H, CH_2_-C**H_2_**-CH_3_), 0.98 (t, *J* = 7.4 Hz, 3H, CH_2_-C**H_3_**). *Overlapping Signals.

^13^C{^1^H} NMR (101 MHz):	162.83 (d, *J* = 259.1 Hz), 161.67 (dd, *J* = 258.7, 6.3 Hz), 159.55 (dd, *J* = 251.3, 9.7 Hz), 158.95, 151.42 (t, *J* = 14.4 Hz), 148.36 (t, *J* = 10.4 Hz), 144.72, 135.90 (d, *J* = 8.6 Hz), 134.72, 133.27, 129.44, 126.89, 118.51 (d, *J* = 20.9 Hz), 118.40, 113.70 (d, *J* = 8.2 Hz), 110.53 (dd, *J* = 23.1, 3.0 Hz), 107.18 – 106.16 (m), 101.34 (d, *J* = 15.1 Hz), 37.72, 24.43, 13.81.

^19^F NMR (376 MHz):	-106.08 (t, *J_F-H_* = 8.1 Hz, 1F, Ar-**F**), -107.88 (d, *J_F-H_* = 10.7 Hz, 2F, Ar-**F**), -111.88 (d, *J_F-H_* = 9.1 Hz, 2F, Ar-**F**).

**Fig. S66**	^1^H NMR spectra of **NC1220** in CDCl_3_**.**

**Fig. S67**	^13^C{^1^H} NMR spectra of **NC1220** in CDCl_3_**.**

**Fig. S68**	^19^F NMR spectra of **NC1220** in CDCl_3_**.**

**NC2220**

*4'-cyano-2,3',5',6-tetrafluoro-[1,1'-biphenyl]-4-yl 3,5-difluoro-4'-propyl-[1,1'-biphenyl]-4-carboxylate*

Yield:	(white crystalline solid) 190 mg, 72 %

Re-crystallisation solvent:	MeCN

^1^H NMR (400 MHz):	7.53 (ddd, *J* = 8.2, 1.9, 1.6 Hz, 2H, Ar-**H**), 7.32 (d, *J* = 8.3 Hz, 2H, Ar-**H**), 7.30 – 7.25 (m, 2H, Ar-**H**)^†^, 7.22 (d, *J* = 8.8 Hz, 2H, Ar-**H**), 7.09 (ddd, *J* = 8.2, 5.7, 3.2 Hz, 2H, Ar-**H**), 2.66 (t, *J* = 7.5 Hz, 2H, Ar-C**H_2_**-CH_2_), 1.69 (h, *J* = 7.5 Hz, 2H, CH_2_-C**H_2_**-CH_3_), 0.98 (t, *J* = 7.3 Hz, 3H, CH_2_-C**H_3_**). ^†^ Overlapping CDCl_3_.

^13^C{^1^H} NMR (101 MHz):	162.90 (dd, *J* = 261.4, 4.4 Hz), 160.85 (dd, *J* = 259.1, 8.1 Hz), 159.27 (dd, *J* = 252.6, 8.6 Hz), 158.85, 151.86 (t, *J* = 14.3 Hz), 148.45 (t, *J* = 10.5 Hz), 144.75, 136.68 (t, *J* = 10.7 Hz), 134.68, 129.45, 126.89, 114.28 (dd, *J* = 20.6, 2.6 Hz), 113.06 (t, *J* = 17.6 Hz), 110.55 (dd, *J* = 23.1, 3.1 Hz), 108.96, 107.19 – 106.61 (m), 92.28 (t, *J* = 19.2 Hz), 37.72, 24.43, 13.81.

^19^F NMR (376 MHz):	-103.62 (d, *J_F-H_* = 9.0 Hz, 2F, Ar-**F**), -107.81 (d, *J_F-H_* = 10.9 Hz, 2F, Ar-**F**), -111.57 (d, *J_F-H_* = 9.4 Hz, 2F, Ar-**F**).

**Fig. S69**	^1^H NMR spectra of **NC2220** in CDCl_3_**.**

**Fig. S70**	^13^C{^1^H} NMR spectra of **NC2220** in CDCl_3_**.**

**Fig. S71**	^19^F NMR spectra of **NC2220** in CDCl_3_**.**

**NC2121**

*4'-cyano-2,3',5'-trifluoro-[1,1'-biphenyl]-4-yl 2',3,5-trifluoro-4'-propyl-[1,1'-biphenyl]-4-carboxylate*

Yield:	(white solid) 158 mg, 60 %

Re-crystallisation solvent:	MeCN

^1^H NMR (400 MHz):	7.50 (t, *J* = 8.6 Hz, 1H, Ar-**H**), 7.37 (t, *J* = 8.0 Hz, 1H, Ar-**H**), 7.31 – 7.20 (m, 6H, Ar-**H**)^†^, 7.09 (dd, *J* = 7.9, 1.7 Hz, 1H, Ar-**H**), 7.03 (dd, *J* = 12.0, 1.6 Hz, 1H, Ar-**H**), 2.65 (t, *J* = 7.4 Hz, 2H, Ar-C**H_2_**-CH_2_), 1.69 (h, *J* = 7.4 Hz, 2H, CH_2_-C**H_2_**-CH_3_), 0.98 (t, *J* = 7.3 Hz, 3H, CH_2_-C**H_3_**). ^†^Overlapping CDCl_3_.

^13^C{^1^H} NMR (101 MHz):	162.83 (dd, *J* = 261.0, 4.7 Hz), 160.47 (dd, *J* = 258.8, 5.6 Hz), 159.15 (d, *J* = 2.9 Hz), 158.30 (dt, *J* = 252.7, 9.2 Hz), 152.15 (d, *J* = 10.9 Hz), 147.10 (d, *J* = 8.1 Hz), 143.24 – 142.58 (m), 130.63 (d, *J* = 3.6 Hz), 129.74 (d, *J* = 2.9 Hz), 125.06 (d, *J* = 3.0 Hz), 123.24 (d, *J* = 12.4 Hz), 122.64 (d, *J* = 12.4 Hz), 118.58 (d, *J* = 3.7 Hz), 116.48 (d, *J* = 22.0 Hz), 112.66 (m), 111.02 (d, *J* = 25.9 Hz), 109.11, 107.76 (t, *J* = 17.4 Hz), 91.54 (t, *J* = 18.9 Hz), 37.55, 24.13, 13.72.

^19^F NMR (376 MHz):	-103.44 (d, *J_F-H_* = 9.2 Hz, 2F, Ar-**F**), -108.64 (d, *J_F-H_* = 10.7 Hz, 2F, Ar-**F**), -113.17 (t, *J_F-H_* = 9.7 Hz, 1F, Ar-**F**), -117.55 (t, *J_F-H_* = 9.4 Hz, 1F, Ar-**F**).

**Fig. S72**	^1^H NMR spectra of **NC2121** in CDCl_3_**.**

**Fig. S73**	^13^C{^1^H} NMR spectra of **NC2121** in CDCl_3_**.**

**Fig. S74**	^19^F NMR spectra of **NC2121** in CDCl_3_**.**

**NC1221**

*4'-cyano-2,3',6-trifluoro-[1,1'-biphenyl]-4-yl 2',3,5-trifluoro-4'-propyl-[1,1'-biphenyl]-4-carboxylate*

Yield:	(white crystalline solid) 171 mg, 65 %

Re-crystallisation solvent:	MeCN

^1^H NMR (400 MHz):	7.73 (t, *J* = 7.3 Hz, 1H, Ar-**H**), 7.38 (m, 3H, Ar-**H**)^*^, 7.27 (d, *J* = 10.9 Hz, 2H, Ar-**H**)^†^, 7.12 – 6.99 (m, 4H, Ar-**H**)^*^, 2.65 (t, *J* = 7.6 Hz, 2H, Ar-C**H_2_**-CH_2_), 1.69 (h, *J* = 7.4 Hz, 2H, CH_2_-C**H_2_**-CH_3_), 0.98 (t, *J* = 7.3 Hz, 3H, CH_2_-C**H_3_**). *Overlapping signals, ^†^ Overlapping CDCl_3_.

^13^C{^1^H} NMR (101 MHz):	162.64 (d, *J* = 259.3 Hz), 160.50 (dd, *J* = 258.4, 5.8 Hz), 159.85, 158.84 (dd, *J* = 251.4, 8.7 Hz), 158.34 (d, *J* = 250.2 Hz), 151.30 (t, *J* = 14.4 Hz), 147.17 (d, *J* = 7.9 Hz), 143.02 (d, *J* = 11.0 Hz), 135.83 (d, *J* = 8.4 Hz), 129.74 (d, *J* = 3.0 Hz), 125.08 (d, *J* = 3.1 Hz), 122.66 (d, *J* = 12.0 Hz), 118.51 (d, *J* = 20.7 Hz), 116.49 (d, *J* = 22.0 Hz), 113.70 (d, *J* = 13.4 Hz), 112.76 (dt, *J* = 23.1, 3.6 Hz), 107.46 (t, *J* = 16.6 Hz), 106.74 (m), 101.35 (d, *J* = 15.5 Hz), 37.55, 24.13, 13.72.

^19^F NMR (376 MHz):	-106.07 (t, *J_F-H_* = 8.2 Hz, 1F, Ar-**F**), -108.44 (d, *J_F-H_* = 10.6 Hz, 2F, Ar-**F**), -111.83 (d, *J_F-H_* = 9.0 Hz, 2F, Ar-**F**), -117.52 (t, *J_F-H_* = 9.4 Hz, 1F, Ar-**F**).

**Fig. S75**	^1^H NMR spectra of **NC1221** in CDCl_3_**.**

**Fig. S76**	^13^C{^1^H} NMR spectra of **NC1221** in CDCl_3_**.**

**Fig. S77**	^19^F NMR spectra of **NC1221** in CDCl_3_**.**

**NC2221**

*4'-cyano-2,3',5',6-tetrafluoro-[1,1'-biphenyl]-4-yl 2',3,5-trifluoro-4'-propyl-[1,1'-biphenyl]-4-carboxylate*

Yield:	(white needles) 136 mg, 50 %

Re-crystallisation solvent:	MeCN

^1^H NMR (400 MHz):	7.37 (t, *J* = 8.0 Hz, 1H, Ar-**H**), 7.31 – 7.19 (m, 4H, Ar-**H**)^*†^, 7.13 – 7.06 (m, 3H, Ar-**H**)^*^, 7.04 (dd, *J* = 11.9, 1.6 Hz, 1H, Ar-**H**), 2.65 (t, *J* = 7.3 Hz, 2H, Ar-C**H_2_**-CH_2_), 1.69 (h, *J* = 7.4 Hz, 2H, CH_2_-C**H_2_**-CH_3_), 0.98 (t, *J* = 7.3 Hz, 3H, CH_2_-C**H_3_**).^*^ Overlapping Signals, ^†^ Overlapping CDCl_3_.

^13^C{^1^H} NMR (101 MHz):	162.73 (dd, *J* = 262.0, 5.1 Hz), 160.38 (dd, *J* = 258.9, 6.4 Hz), 160.96 – 158.14 (m)* 158.75, 151.81 (t, *J* = 14.4 Hz), 147.20 (d, *J* = 7.9 Hz), 143.11 (t, *J* = 10.9 Hz), 136.60 (t, *J* = 10.7 Hz), 129.73 (d, *J* = 3.1 Hz), 125.08 (d, *J* = 3.1 Hz), 122.63 (d, *J* = 12.5 Hz), 116.50 (d, *J* = 22.0 Hz), 114.28 (dd, *J* = 21.0, 3.0 Hz), 113.37 – 112.54 (m), 108.95, 107.34 (t, *J* = 16.6 Hz), 107.15 – 106.58 (m), 92.19 (t, *J* = 19.2 Hz). ^*^ Overlapping Signals.

^19^F NMR (376 MHz):	-103.61 (d, *J_F-H_* = 9.1 Hz, 2F, Ar-**H**), -108.37 (d, *J_F-H_* = 10.6 Hz, 2F, Ar-**F**), -111.51 (d, *J_F-H_* = 9.4 Hz, 2F, Ar-**F**), -117.51 (t, *J_F-H_* = 10.0 Hz, 1F, Ar-**F**).

**Fig. S78**	^1^H NMR spectra of **NC2221** in CDCl_3_**.**

**Fig. S79**	^13^C{^1^H} NMR spectra of **NC2221** in CDCl_3_**.**

**Fig. S80**	^19^F NMR spectra of **NC2221** in CDCl_3_**.**

**NC2122**

*4'-cyano-2,3',5'-trifluoro-[1,1'-biphenyl]-4-yl 2',3,5,6'-tetrafluoro-4'-propyl-[1,1'-biphenyl]-4-carboxylate*

Yield:	(white crystalline solid) 163 mg, 60 %

Re-crystallisation solvent:	MeCN

^1^H NMR (400 MHz):	7.51 (t, *J* = 8.6 Hz, 1H, Ar-**H**), 7.32 – 7.16 (m, 7H, Ar-**H**)^*†^, 6.87 (d, *J* = 9.1 Hz, 2H, Ar-**H**), 2.64 (t, *J* = 7.6 Hz, 2H, Ar-C**H_2_**-CH_2_), 1.69 (h, *J* = 7.4 Hz, 2H, CH_2_-C**H_2_**-CH_3_), 0.99 (t, *J* = 7.3 Hz, 3H, CH_2_-C**H_3_**). ^*^Overlapping Signals^, †^ Overlapping CDCl_3_.

^13^C{^1^H} NMR (101 MHz):	162.75 (dd, *J* = 260.9, 5.0 Hz), 160.23 (dd, *J* = 257.8, 6.8 Hz), 160.69 – 158.05 (m)^*^, 152.10 (d, *J* = 11.2 Hz), 147.33 (t, *J* = 9.5 Hz), 142.98 (t, *J* = 9.8 Hz), 136.45 (tz, *J* = 11.3 Hz), 130.65 (d, *J* = 3.7 Hz), 123.36 (d, *J* = 12.5 Hz), 118.56 (d, *J* = 3.8 Hz), 114.45 (dd, *J* = 23.7, 2.1 Hz), 112.60 (dt, *J* = 21.0, 3.9 Hz), 112.24 – 111.74 (m), 111.00 (d, *J* = 25.9 Hz), 108.57, 108.49 (t, *J* = 16.5 Hz), 91.56 (t, *J* = 19.1 Hz), 37.69, 23.85, 13.63. ^*^ Overlapping Signals.

^19^F NMR (376 MHz):	-103.43 (d, *J_F-H_* = 9.2 Hz, 1F, Ar-**F**), -109.11 (d, *J_F-H_* = 9.9 Hz, 1F, Ar-**F**), -113.12 (t, *J_F-H_* = 9.8 Hz, 1F, Ar-**F**), -114.95 (d, *J_F-H_* = 9.7 Hz, 1F, Ar-**F**).

**Fig. S81**	^1^H NMR spectra of **NC2122** in CDCl_3_.

**Fig. S82**	^13^C{^1^H} NMR spectra of **NC2122** in CDCl_3_.

**Fig. S83**	^19^F NMR spectra of **NC2122** in CDCl_3_**.**

**NC1222**

*4'-cyano-2,3',6-trifluoro-[1,1'-biphenyl]-4-yl 2',3,5,6'-tetrafluoro-4'-propyl-[1,1'-biphenyl]-4-carboxylate*

Yield:	(white crystalline solid) 180 mg, 55 %

Re-crystallisation solvent:	MeCN

^1^H NMR (400 MHz):	7.73 (t, *J* = 8.2 Hz, 1H, Ar-**H**), 7.40 (t, *J* = 8.1 Hz, 2H, Ar-**H**), 7.20 (d, *J* = 10.7 Hz, 2H, Ar-**H**), 7.07 (ddd, *J* = 8.1, 6.2, 3.1 Hz, 2H, Ar-**H**), 6.87 (ddd, *J* = 9.1, 5.1, 2.9 Hz, 2H, Ar-**H**), 2.63 (t, *J* = 7.6 Hz, 2H, Ar-C**H_2_**-CH_2_), 1.68 (h, *J* = 7.0 Hz, 2H, CH_2_-C**H_2_**-CH_3_), 0.98 (t, *J* = 7.3 Hz, 3H, CH_2_-C**H_3_**).

^13^C{^1^H} NMR (101 MHz):	162.60 (d, *J* = 259.8 Hz), 160.40 (dd, *J* = 258.9, 6.1 Hz), 159.56, 158.55 (dd, *J* = 251.1, 8.0 Hz), 158.16 (dd, *J* = 250.4, 6.6 Hz), 151.32 (t, *J* = 14.8 Hz), 147.39 (t, *J* = 10.1 Hz), 136.62 (t, *J* = 11.6 Hz), 135.86 (d, *J* = 8.9 Hz), 133.28, 126.88 (d, *J* = 2.2 Hz), 118.51 (d, *J* = 21.3 Hz), 114.53 (dd, *J* = 23.1, 2.9 Hz), 112.02 (dd, *J* = 24.6, 5.9 Hz), 108.26 (d, *J* = 16.8 Hz), 107.02 – 106.31 (m), 101.35 (d, *J* = 15.3 Hz), 37.69, 23.84, 13.62.

^19^F NMR (376 MHz):	-106.07 (t, *J_F-H_* = 8.2 Hz, 1F, Ar-**F**), -108.92 (d, *J_F-H_* = 10.3 Hz, 2F, Ar-**F**), -111.77 (d, *J_F-H_* = 9.0 Hz, 2F, Ar-**F**), -114.94 (d, *J_F-H_* = 9.8 Hz, 2F, Ar-**F**).

**Fig. S84**	^1^H NMR spectra of **NC1222** in CDCl_3_**.**

**Fig. S85**	^13^C{^1^H} NMR spectra of **NC1222** in CDCl_3_**.**

**Fig. S86**	^19^F NMR spectra of **NC1222** in CDCl_3_**.**

**NC2222**

*4'-cyano-2,3',5',6-tetrafluoro-[1,1'-biphenyl]-4-yl 2',3,5,6'-tetrafluoro-4'-propyl-[1,1'-biphenyl]-4-carboxylate*

Yield:	(white needles) 135 mg, 48 %

Re-crystallisation solvent:	MeCN

^1^H NMR (400 MHz):	7.25 – 7.17 (m, 4H, Ar-**H**)^*^, 7.09 (ddd, *J* = 8.2, 5.7, 2.9 Hz, 2H, Ar-**H**), 6.87 (ddd, *J* = 9.1, 5.0, 2.8 Hz, 2H, Ar-**H**), 2.64 (t, *J* = 7.4 Hz, 2H, Ar-C**H_2_**-CH_2_), 1.69 (h, *J* = 7.4 Hz, 2H, CH_2_-C**H_2_**-CH_3_), 0.99 (t, *J* = 7.3 Hz, 3H, CH_2_-C**H_3_**). *Overlapping Signals.

^13^C{^1^H} NMR (126 MHz):	163.02 (dd, *J* = 256.7, 4.6 Hz), 161.97 (dd, *J* = 255.5, 2.9 Hz), 160.74 (dd, *J* = 252.2, 8.3 Hz), 159.08 (dd, *J* = 250.0, 6.8 Hz), 151.90 (t, *J* = 14.4 Hz), 147.56 (t, *J* = 9.6 Hz), 137.21 – 136.48 (m), 114.89 – 114.24 (m), 113.21 (t, *J* = 17.6 Hz), 112.43 (tj, *J* = 17.8 Hz), 112.16 (dd, *J* = 20.5, 4.9 Hz), 108.28 (t, *J* = 16.4 Hz), 107.25 – 106.82 (m), 92.44 (t, *J* = 19.2 Hz), 37.83, 23.98, 13.77.

^19^F NMR (376 MHz):	-103.61 (d, *J_F-H_* = 8.8 Hz, 2F, Ar-**F**), -108.85 (d, *J_F-H_* = 10.2 Hz, 2F, Ar-**F**), -111.46 (d, *J_F-H_* = 9.4 Hz, 2F, Ar-**F**), -114.94 (d, *J_F-H_* = 9.7 Hz, 2F, Ar-**F**).

**Fig. S87**	^1^H NMR spectra of **NC2222** in CDCl_3_**.**

**Fig. S88**	^13^C{^1^H} NMR spectra of **NC2222** in CDCl_3_**.**

**Fig. S89**	^19^F NMR spectra of **NC2222** in CDCl_3_**.**

**NC0000**

*4'-cyano-[1,1'-biphenyl]-4-yl 4'-propyl-[1,1'-biphenyl]-4-carboxylate*

Yield:	(white solid) 175 mg, 84 %

Re-crystallisation solvent:	EtOH

^1^H NMR (400 MHz):	8.28 (d, *J* = 8.2 Hz, 2H, Ar-**H**), 7.79 – 7.62 (m, 8H, Ar-**H**)^*†^, 7.60 (d, *J* = 7.8 Hz, 2H, Ar-**H**), 7.37 (d, *J* = 8.7 Hz, 2H, Ar-**H**), 7.31 (d, *J* = 7.9 Hz, 2H, Ar-**H**), 2.67 (t, *J* = 7.6 Hz, 2H, Ar-C**H_2_**-CH_2_), 1.71 (h, *J* = 7.5 Hz, 2H, CH_2_-C**H_2_**-CH_3_), 1.00 (t, *J* = 7.3 Hz, 3H, CH_2_-C**H_3_**).

^13^C{^1^H} NMR (101 MHz): 165.05, 151.52, 146.56, 144.83, 143.29, 137.08, 136.90, 132.69, 130.78, 129.19, 128.43, 127.73, 127.62, 127.18, 127.08, 122.54, 118.90, 111.08, 37.75, 24.54, 13.89.

**Fig. S90**	^1^H NMR spectra of **NC0000** in CDCl_3_**.**

**Fig. S91**	^13^C{^1^H} NMR spectra of **NC0000** in CDCl_3_**.**

**NC1000**

*4'-cyano-3'-fluoro-[1,1'-biphenyl]-4-yl 4'-propyl-[1,1'-biphenyl]-4-carboxylate*

Yield:	(white solid) 174 mg, 80 %

Re-crystallisation solvent:	EtOH

^1^H NMR (400 MHz):	8.27 (ddd, *J* = 8.5, 1.9, 1.8 Hz, 2H, Ar-**H**), 7.75 (ddd, *J* = 8.6, 2.0, 1.9 Hz, 2H, Ar-**H**), 7.70 (dd, *J* = 6.7, 1.4 Hz, 1H, Ar-**H**), 7.65 (ddd, *J* = 8.7, 2.1, 2.1 Hz, 2H, Ar-**H**), 7.59 (ddd, *J* = 8.2, 2.2, 1.8 Hz, 2H, Ar-**H**), 7.50 (dd, *J* = 8.0, 1.5 Hz, 1H, Ar-**H**), 7.44 (dd, *J* = 10.1, 1.6 Hz, 1H, Ar-**H**), 7.38 (ddd, *J* = 8.7, 2.8, 2.1 Hz, 2H, Ar-**H**), 7.31 (d, *J* = 8.2 Hz, 2H, Ar-**H**), 2.66 (t, *J* = 7.3 Hz, 2H, Ar-C**H_2_**-CH_2_), 1.70 (h, *J* = 7.5 Hz, 2H, CH_2_-C**H_2_**-CH_3_), 0.99 (t, *J* = 7.3 Hz, 3H, CH_2_-C**H_3_**).

^13^C{^1^H} NMR (101 MHz):	164.97, 163.49 (d, *J* = 258.8 Hz), 151.98, 147.69 (d, *J* = 7.9 Hz), 146.64, 143.31, 137.05, 135.72 (d, *J* = 1.4 Hz), 130.79, 129.19, 128.43, 127.50, 127.17, 127.09, 123.38 (d, *J* = 3.2 Hz), 122.72, 114.82 (d, *J* = 20.2 Hz), 114.04, 99.98 (d, *J* = 15.9 Hz), 37.74, 24.54, 13.88.

^19^F NMR (376 MHz):	-105.93 (t, *J_F-H_* = 8.4 Hz, 1F, Ar-**F**).

**Fig. S92**	^1^H NMR spectra of **NC1000** in CDCl_3_.

**Fig. S93**	^13^C{^1^H} NMR spectra of **NC1000** in CDCl_3_.

**Fig. S94**	^19^F NMR spectra of **NC1000** in CDCl_3_.

**NC0100**

*4'-cyano-2-fluoro-[1,1'-biphenyl]-4-yl 4'-propyl-[1,1'-biphenyl]-4-carboxylate*

Yield:	(white solid) 170 mg, 78 %

Re-crystallisation solvent:	EtOH

^1^H NMR (400 MHz):	8.26 (ddd, *J* = 8.6, 1.9, 1.9 Hz, 2H, Ar-**H**), 7.79 – 7.72 (m, 4H, Ar-**H**)^*^, 7.70 – 7.65 (m, 2H, Ar-**H**)^*^, 7.59 (ddd, *J* = 8.2, 2.0, 1.8 Hz, 2H, Ar-**H**), 7.50 (t, *J* = 8.6 Hz, 1H, Ar-**H**), 7.31 (ddd, *J* = 8.3, 1.9, 1.8 Hz, 2H, Ar-**H**), 7.21 – 7.15 (m, 2H, Ar-**H**)^*^, 2.67 (t, *J* = 7.3 Hz, 2H, Ar-C**H_2_**-CH_2_), 1.70 (h, *J* = 7.4 Hz, 2H, CH_2_-C**H_2_**-CH_3_), 0.99 (t, *J* = 7.3 Hz, 3H, CH_0_-C**H_3_**).

^13^C{^1^H} NMR (101 MHz):	164.65, 160.86, 158.36, 152.04 (d, *J* = 11.0 Hz), 146.79, 143.37, 139.81, 136.99, 132.34, 130.93 – 130.77 (m), 129.67 (d, *J* = 3.3 Hz), 129.21, 127.18, 127.13, 124.88 (d, *J* = 13.1 Hz), 118.39 (d, *J* = 3.6 Hz), 118.37, 111.57, 110.81 (d, *J* = 25.8 Hz), 37.75, 24.53, 13.88.

^19^F NMR (376 MHz):	-114.39 (t, *J_F-H_* = 9.7 Hz, 1F, Ar-**F**).

**Fig. S95**	^1^H NMR spectra of **NC0100** in CDCl_3_.

**Fig. S96**	^13^C{^1^H} NMR spectra of **NC0100** in CDCl_3_.

**Fig. S97**	^19^F NMR spectra of **NC0100** in CDCl_3_.

**NC0010**

*4'-cyano-[1,1'-biphenyl]-4-yl 3-fluoro-4'-propyl-[1,1'-biphenyl]-4-carboxylate*

Yield:	(white solid) 170 mg, 78 %

Re-crystallisation solvent:	EtOH

^1^H NMR (400 MHz):	8.17 (t, *J* = 7.9 Hz, 1H, Ar-**H**), 7.77 – 7.68 (m, 4H, Ar-**H**)^*^, 7.66 (ddd, *J* = 8.7, 2.8, 2.0 Hz, 2H, Ar-**H**), 7.57 (ddd, *J* = 8.2, 2.0, 1.8 Hz, 2H, Ar-**H**), 7.52 (dd, *J* = 8.2, 1.7 Hz, 1H, Ar-**H**), 7.45 (dd, *J* = 12.1, 1.7 Hz, 1H, Ar-**H**), 7.38 (ddd, *J* = 8.5, 2.8, 2.0 Hz, 2H, Ar-**H**), 7.31 (ddd, *J* = 8.2, 2.3, 1.5 Hz, 2H, Ar-**H**), 2.66 (t, *J* = 7.5 Hz, 2H, Ar-C**H_2_**-CH_2_), 1.70 (h, *J* = 7.4 Hz, 2H, CH_2_-C**H_2_**-CH_3_), 0.99 (t, *J* = 7.3 Hz, 3H, CH_2_-C**H_3_**). ^*^ Overlapping Signals.

^13^C{^1^H} NMR (126 MHz):	163.80 (d, *J* = 261.7 Hz), 162.64 (d, *J* = 4.3 Hz), 151.17, 148.83 (d, *J* = 9.0 Hz), 144.82, 144.01, 137.05, 135.74, 132.99, 132.69, 129.31, 128.43, 127.75, 127.06, 122.50, 122.50 (d, *J* = 1.5 Hz), 118.89, 115.76 (d, *J* = 9.5 Hz), 115.27 (d, *J* = 23.1 Hz), 111.09, 37.74, 24.49, 13.85.

^19^F NMR (376 MHz):	-107.56 (dd, *J_F-H_* = 12.2 Hz, *J_F-H_* = 7.6 Hz, 1F, Ar-**F**).

**Fig. S98**	^1^H NMR spectra of **NC0010** in CDCl_3_**.**

**Fig. S99**	^13^C{^1^H} NMR spectra of **NC0010** in CDCl_3_.

**Fig. S100**	^19^F NMR spectra of **NC0010** in CDCl_3_**.**

**NC1010**

*4'-cyano-3'-fluoro-[1,1'-biphenyl]-4-yl 3-fluoro-4'-propyl-[1,1'-biphenyl]-4-carboxylate*

Yield:	(white solid) 156 mg, 69 %

Re-crystallisation solvent:	EtOH

^1^H NMR (400 MHz):	8.19 (t, *J* = 7.9 Hz, 1H, Ar-**H**), 7.73 (dd, *J* = 8.1, 6.6 Hz, 1H, Ar-**H**), 7.67 (ddd, *J* = 8.7, 2.8, 2.1 Hz, 2H, Ar-**H**), 7.60 (ddd, *J* = 8.2, 2.2, 1.6 Hz, 2H, Ar-**H**), 7.57 – 7.44 (m, 4H, Ar-**H**)^*^, 7.42 (ddd, *J* = 8.7, 2.9, 2.0 Hz, 2H, Ar-**H**), 7.34 (ddd, *J* = 8.1, 2.2, 1.8 Hz, 2H, Ar-**H**), 2.69 (t, *J* = 7.6 Hz, 2H, Ar-C**H_2_**-CH_2_), 1.72 (h, *J* = 7.4 Hz, 2H, CH_2_-C**H_2_**-CH_3_), 1.01 (t, *J* = 7.3 Hz, 3H, CH_2_-C**H_3_**). ^*^ Overlapping Signals.

^13^C{^1^H} NMR (126 MHz):	163.60 (d, *J* = 258.4 Hz), 162.55 (d, *J* = 4.2 Hz), 161.73 (d, *J* = 262.0 Hz), 151.63, 148.91 (d, *J* = 8.9 Hz), 147.65 (d, *J* = 8.2 Hz), 144.04, 135.78 (dd, *J* = 16.5, 1.9 Hz)z, 133.84, 132.99, 129.32, 128.43, 127.06, 123.39 (d, *J* = 3.1 Hz), 122.69, 122.49 (d, *J* = 3.2 Hz), 115.64 (d, *J* = 9.6 Hz), 115.28 (d, *J* = 23.1 Hz), 114.84 (d, *J* = 20.3 Hz), 114.03, 100.00 (d, *J* = 15.7 Hz), 37.74, 24.49, 13.85.

^19^F NMR (376 MHz):	-105.93 (t, *J_F-H_* = 7.4 Hz, 1F, Ar-**F**), -107.50 (dd, *J_F-H_* = 12.3 Hz, *J_F-H_* = 7.6 Hz, 1F, Ar-**F**).

**Fig. S101**	^1^H NMR spectra of **NC1010** in CDCl_3._

**Fig. S102**	^13^C{^1^H} NMR spectra of **NC1010** in CDCl_3._

**Fig. S103**	^19^F NMR spectra of **NC1010** in CDCl_3._

**NC0110**

*4'-cyano-2-fluoro-[1,1'-biphenyl]-4-yl 3-fluoro-4'-propyl-[1,1'-biphenyl]-4-carboxylate*

Yield:	(white crystalline solid) 175 mg, 77 %

Re-crystallisation solvent:	EtOH

^1^H NMR (400 MHz):	8.15 (t, *J* = 7.9 Hz, 1H, Ar-**H**), 7.76 (ddd, *J* = 8.0, 2.2, 1.3 Hz, 2H, Ar-**H**), 7.70 – 7.64 (m, 2H, Ar-**H**), 7.57 (ddd, *J* = 8.1, 2.0, 1.7 Hz, 2H, Ar-**H**), 7.54 – 7.47 (m, 2H, Ar-**H**)^*^, 7.45 (dd, *J* = 12.1, 1.7 Hz, 1H, Ar-**H**), 7.31 (ddd, *J* = 8.1, 2.0, 1.8 Hz, 2H, Ar-**H**), 7.23 – 7.16 (m, 2H, Ar-**H**)^*^, 2.66 (t, *J* = 7.4 Hz, 2H, Ar-C**H_2_**-CH_2_), 1.70 (h, *J* = 7.4 Hz, 2H, CH_2_-C**H_2_**-CH_3_), 0.98 (t, *J* = 7.3 Hz, 3H, CH_2_-C**H_3_**). ^*^ Overlapping Signals.

^13^C{^1^H} NMR (126 MHz):	162.79 (d, *J* = 261.8 Hz), 162.22 (d, *J* = 4.2 Hz), 159.58 (d, *J* = 251.3 Hz), 151.64 (d, *J* = 11.1 Hz), 149.09 (d, *J* = 9.0 Hz), 144.10, 139.77, 135.65, 133.00, 132.35, 130.87 (d, *J* = 4.2 Hz), 129.67 (d, *J* = 3.3 Hz), 129.33, 127.07, 125.03 (d, *J* = 13.2 Hz), 122.53 (d, *J* = 3.2 Hz), 118.75, 118.35 (d, *J* = 3.6 Hz), 115.40, 115.31 (d, *J* = 23.0 Hz), 111.59, 110.78 (d, *J* = 25.9 Hz), 37.74, 24.49, 13.85.

^19^F NMR (376 MHz):	-107.30 (dd, *J_F-H_* = 12.3 Hz, *J_F-H_* = 7.6 Hz, 1F, Ar-**F**), -114.36 (t, *J_F-H_* = 9.7 Hz, 1F, Ar-**F**).

**Fig. S104**	^1^H NMR spectra of **NC0110** in CDCl_3_.

**Fig. S105**	^13^C{^1^H} NMR spectra of **NC0110** in CDCl_3_.

**Fig. S106**	^19^F NMR spectra of **NC0110** in CDCl_3_.

**NC0020**

*4'-cyano-[1,1'-biphenyl]-4-yl 3,5-difluoro-4'-propyl-[1,1'-biphenyl]-4-carboxylate*

Yield:	(white solid) 134 mg, 60 %

Re-crystallisation solvent:	EtOH

^1^H NMR (400 MHz):	7.77 – 7.63 (m, 4H, Ar-**H**)*, 7.53 (ddd, *J* = 8.3, 1.9, 1.9 Hz, 2H, Ar-**H**), 7.40 (ddd, *J* = 8.9, 2.8, 2.1 Hz, 2H, Ar-**H**), 7.32 (ddd, *J* = 8.2, 1.6, 1.5 Hz, 2H, Ar-**H**), 7.26 (ddd, *J* = 9.4, 3.4, 2.1 Hz, 2H, Ar-**H**), 2.66 (t, *J* = 7.2 Hz, 2H, Ar-C**H_2_**-CH_2_), 1.69 (h, *J* = 7.5 Hz, 2H, CH_2_-C**H_2_**-CH_3_), 0.98 (t, *J* = 7.3 Hz, 3H, CH_2_-C**H_3_**). ^* ­^Overlapping Signals.

^13^C{^1^H} NMR (101 MHz):	161.52 (dd, *J* = 257.6, 6.6 Hz), 159.88, 150.87, 147.66 (t, *J* = 9.8 Hz), 144.73, 144.49, 137.32, 134.93, 132.69, 129.39, 128.48, 127.77, 126.89, 122.39, 118.86, 111.17, 110.43 (dd, *J* = 23.4, 2.8 Hz), 107.83 (t, *J* = 15.7 Hz), 37.73, 24.45, 13.82.

^19^F NMR (376 MHz):	-108.53 (d, *J_F-H_* = 10.4 Hz, 2F, Ar-**F**).

**Fig. S107**	^1^H NMR spectra of **NC0020** in CDCl_3_**.**

**Fig. S108**	^13^C{^1^H} NMR spectra of **NC0020** in CDCl_3_**.**

**Fig. S109**	^19^F NMR spectra of **NC0020** in CDCl_3_**.**

**NC1020**

*4'-cyano-3'-fluoro-[1,1'-biphenyl]-4-yl 3,5-difluoro-4'-propyl-[1,1'-biphenyl]-4-carboxylate*

Yield:	(white solid) 172 mg, 73 %

Re-crystallisation solvent:	EtOH

^1^H NMR (400 MHz):	7.70 (dd, *J* = 8.1, 6.6 Hz, 1H, Ar-**H**), 7.65 (ddd, *J* = 8.6, 2.8, 1.9 Hz, 2H, Ar-**H**), 7.53 (ddd, *J* = 8.3, 1.8, 1.8 Hz, 2H, Ar-**H**), 7.49 (dd, *J* = 8.1, 1.6 Hz, 1H, Ar-**H**), 7.46 – 7.39 (m, 3H, Ar-**H**)^*^, 7.31 (ddd, *J* = 8.2, 2.0 Hz, 2H, Ar-**H**), 7.26 (d, *J* = 9.4 Hz, 2H, Ar-**H**)^†^, 2.66 (t, *J* = 7.4 Hz, 2H, Ar-C**H_2_**-CH_2_), 1.69 (h, *J* = 7.5 Hz, 2H, CH_2_-C**H_2_**-CH_3_), 0.98 (t, *J* = 7.3 Hz, 3H, CH_2_-C**H_3_**). *Overlapping Signals, ^†^Overlapping CDCl_3_.

^13^C{^1^H} NMR (101 MHz):	163.28 (d, *J* = 259.4 Hz), 160.23 (dd, *J* = 258.0, 6.4 Hz), 159.79, 151.32, 147.97 – 147.46 (m), 144.52, 136.13, 134.91, 133.86, 129.40, 128.48, 126.89, 123.41 (d, *J* = 3.1 Hz), 122.58, 114.87 (d, *J* = 20.4 Hz), 114.00, 110.45 (dd, *J* = 23.7, 2.1 Hz), 107.70 (t, *J* = 17.6 Hz), 100.08 (d, *J* = 15.7 Hz), 88.00 (t, *J* = 17.6 Hz), 37.72, 24.44, 13.82.

^19^F NMR (376 MHz):	-105.88 (dd, *J_F-H_* = 9.1 Hz, *J_F-H_* = 7.0 Hz, 1F, Ar-**F**), -108.46 (d, *J* = 10.5 Hz, 2F, Ar-**F**).

**Fig. S110**	^1^H NMR spectra of **NC1020** in CDCl_3_.

**Fig. S111**	^13^C{^1^H} NMR spectra of **NC1020** in CDCl_3_.

**Fig. S112**	^19^F NMR spectra of **NC1020** in CDCl_3_.

**NC0120**

*4'-cyano-2-fluoro-[1,1'-biphenyl]-4-yl 3,5-difluoro-4'-propyl-[1,1'-biphenyl]-4-carboxylate*

Yield:	(white solid) 146 mg, 62 %

Re-crystallisation solvent:	EtOH

^1^H NMR (400 MHz):	7.80 – 7.63 (m, 4H, Ar-**H**)^*^, 7.57 – 7.46 (m, 3H, Ar-**H**)^*^, 7.35 – 7.18 (m, 6H, Ar-**H**)^*†^, 2.66 (t, *J* = 7.6 Hz, 2H, Ar-C**H_2_**-CH_2_), 1.70 (h, *J* = 7.4 Hz, 2H, CH_2_-C**H_2_**-CH_3_), 0.98 (t, *J* = 7.3 Hz, 3H, CH_2_-C**H_3_**). ^*^Overlapping Signals, ^†^ Overlapping CDCl_3_.

^13^C{^1^H} NMR (101 MHz):	161.44 (dd, *J* = 258.3, 6.6 Hz), 158.89 (d, *J* = 251.7 Hz), 158.31, 151.28 (d, *J* = 11.0 Hz), 147.91 (t, *J* = 10.6 Hz), 144.59, 139.68, 134.84, 132.36, 130.93 (d, *J* = 4.2 Hz), 129.68 (d, *J* = 3.2 Hz), 129.42, 126.89, 125.31 (d, *J* = 13.3 Hz), 118.72, 118.24 (d, *J* = 3.8 Hz), 111.66, 110.95 – 110.18 (m), 107.37 (t, *J* = 16.3 Hz), 37.73, 24.44, 13.82.

^19^F NMR (376 MHz):	-108.23 (d, *J_F-H_* = 10.5 Hz, 2F, Ar-**F**), -114.15 (t, *J_F-H_* = 9.8 Hz, 1F, Ar-**F**).

**Fig. S113**	^1^H NMR spectra of **NC0120** in CDCl_3_**.**

**Fig. S114**	^13^C{^1^H} NMR spectra of **NC0120** in CDCl_3_**.**

**Fig. S115**	^19^F NMR spectra of **NC0120** in CDCl_3_**.**

**NC0021**

*4'-cyano-[1,1'-biphenyl]-4-yl 2',3,5-trifluoro-4'-propyl-[1,1'-biphenyl]-4-carboxylate*

Yield:	(white crystalline solid) 118 mg, 50 %

Re-crystallisation solvent:	EtOH

^1^H NMR (400 MHz):	7.77 – 7.67 (m, 2H, Ar-**H**), 7.66 (ddd, *J* = 8.6, 2.6, 1.9 Hz, 2H, Ar-**H**), 7.41 (ddd, *J* = 8.6, 2.8, 1.9 Hz, 2H, Ar-**H**), 7.37 (t, *J* = 8.2 Hz, 1H, Ar-**H**), 7.29 – 7.23 (m, 2H, Ar-**H**)^†^, 7.09 (dd, *J* = 7.9, 1.6 Hz, 1H, Ar-**H**), 7.03 (dd, *J* = 12.0, 1.6 Hz, 1H, Ar-**H**), 2.65 (t, *J* = 7.6 Hz, 2H, Ar-C**H_2_**-CH_2_), 1.69 (h, *J* = 7.3 Hz, 2H, CH_2_-C**H_2_**-CH_3_), 0.98 (t, *J* = 7.3 Hz, 3H, CH_2_-C**H_3_**).^†^ Overlapping CDCl_3_.

^13^C{^1^H} NMR (101 MHz):	160.96 (dd, *J* = 257.1, 6.8 Hz), 159.78, 158.34 (d, *J* = 250.4 Hz), 158.22, 146.94 (d, *J* = 7.8 Hz), 144.73, 142.30 (d, *J* = 12.1 Hz), 137.37, 132.70, 129.77 (d, *J* = 3.0 Hz), 128.49, 127.77, 125.02 (d, *J* = 3.1 Hz), 122.37, 118.86, 116.45 (d, *J* = 22.0 Hz), 112.65 (dt, *J* = 23.3, 3.3 Hz), 111.18, 37.54, 24.14, 13.72.

^19^F NMR (376 MHz):	-109.08 (d, *J_F-H_* = 10.3 Hz, 2F, Ar-**F**), -117.59 (t, *J_F-H_* = 9.9 Hz, 1F, Ar-**F**).

**Fig. S116**	^1^H NMR spectra of **NC0021** in CDCl_3_**.**

**Fig. S117**	^13^C{^1^H} NMR spectra of **NC0021** in CDCl_3_**.**

**Fig. S118**	^19^F NMR spectra of **NC0021** in CDCl_3_**.**

**NC1021**

*4'-cyano-3'-fluoro-[1,1'-biphenyl]-4-yl 2',3,5-trifluoro-4'-propyl-[1,1'-biphenyl]-4-carboxylate*

Yield:	(white solid) 110 mg, 45 %

Re-crystallisation solvent:	EtOH

^1^H NMR (400 MHz):	7.70 (dd, *J* = 8.1, 6.7 Hz, 1H, Ar-**H**), 7.65 (ddd, *J* = 8.7, 2.7, 2.0 Hz, 2H, Ar-**H**), 7.49 (dd, *J* = 8.1, 1.6 Hz, 1H, Ar-**H**), 7.46 – 7.40 (m, 3H, Ar-**H**)^*^, 7.37 (t, *J* = 8.0 Hz, 1H, Ar-**H**), 7.30 – 7.23 (m, 2H, Ar-**H**)^†^, 7.09 (dd, *J* = 7.9, 1.7 Hz, 1H, Ar-**H**), 7.03 (dd, *J* = 12.0, 1.7 Hz, 1H, Ar-**H**), 2.65 (t, *J* = 7.5 Hz, 2H, Ar-C**H_2_**-CH_2_), 1.69 (h, *J* = 7.4 Hz, 2H. CH_2_-C**H_2_**-CH_3_), 0.98 (t, *J* = 7.3 Hz, 3H, CH_2_-C**H_3_**).

^13^C{^1^H} NMR (101 MHz):	164.77 (d, *J* = 258.9 Hz), 161.20 (dd, *J* = 258.0, 6.7 Hz), 159.01 (d, *J* = 249.9 Hz), 159.68, 151.29, 147.55 (d, *J* = 8.0 Hz), 146.97 (d, *J* = 8.0 Hz), 142.34 (t, *J* = 11.1 Hz), 136.17 (d, *J* = 1.5 Hz), 133.86, 129.77 (d, *J* = 3.0 Hz), 128.49, 125.03 (d, *J* = 3.1 Hz), 123.42 (d, *J* = 3.2 Hz), 122.56, 116.45 (d, *J* = 22.0 Hz), 114.87 (d, *J* = 20.3 Hz), 112.77, 112.66 (dt, *J* = 23.1, 3.4 Hz), 108.33 (t, *J* = 16.8 Hz), 100.09 (d, *J* = 15.6 Hz), 37.54, 24.14, 13.72.

^19^F NMR (376 MHz):	-105.88 (t, *J_F-H_* = 7.4 Hz, 1F, Ar-**F**), -109.00 (d, *J_F-H_* = 10.3 Hz, 2F, Ar-**F**), -117.59 (d, *J_F-H_* = 9.9 Hz, 1F, Ar-**F**).

**Fig. S119**	^1^H NMR spectra of **NC1021** in CDCl_3_**.**

**Fig. S120**	^13^C{^1^H} NMR spectra of **NC1021** in CDCl_3_**.**

**Fig. S121**	^19^F NMR spectra of **NC1021** in CDCl_3_**.**

**NC0121**

*4'-cyano-2-fluoro-[1,1'-biphenyl]-4-yl 2',3,5-trifluoro-4'-propyl-[1,1'-biphenyl]-4-carboxylate*

Yield:	(white solid) 100 mg, 41 %

Re-crystallisation solvent:	EtOH

^1^H NMR (400 MHz):	7.76 (ddd, *J* = 8.7, 1.7, 1.7 Hz, 2H, Ar-**H**), 7.70 – 7.63 (m, 2H, Ar-**H**), 7.50 (t, *J* = 8.6 Hz, 1H, Ar-**H**), 7.37 (t, *J* = 8.0 Hz, 1H, Ar-**H**), 7.30 – 7.18 (m, 4H, Ar-**H**)^*†^, 7.09 (dd, *J* = 8.0, 1.6 Hz, 1H, Ar-**H**), 7.03 (dd, *J* = 11.9, 1.6 Hz, 1H, Ar-**H**), 2.65 (t, *J* = 7.6 Hz, 2H, Ar-C**H_2_**-CH_2_), 1.69 (h, *J* = 7.4 Hz, 2H, CH_2_-C**H_2_**-CH_3_), 0.98 (t, *J* = 7.3 Hz, 3H, CH_2_-C**H_3_**). ^*^ Overlapping Signals, ^†^ Overlapping CDCl_3_.

^13^C{^1^H} NMR (126 MHz):	162.23 (dd, *J* = 257.8, 6.3 Hz), 160.67, 160.28 (dd, *J* = 250.2, 4.4 Hz), 151.35 (d, *J* = 11.0 Hz), 147.16 (d, *J* = 7.9 Hz), 142.73 (t, *J* = 10.7 Hz), 139.78, 132.47, 131.07 (d, *J* = 4.1 Hz), 129.88 (d, *J* = 3.0 Hz), 129.79 (d, *J* = 3.3 Hz), 125.47 (d, *J* = 13.1 Hz), 125.17 (d, *J* = 3.1 Hz), 122.87 (d, *J* = 12.5 Hz), 118.34 (d, *J* = 3.7 Hz), 116.58 (d, *J* = 21.9 Hz), 112.81 (dt, *J* = 23.0, 3.7 Hz), 111.78, 110.79 (d, *J* = 26.1 Hz), 108.12 (t, *J* = 16.7 Hz), 37.66, 24.26, 13.84.

^19^F NMR (376 MHz):	-108.80 (d, *J_F-H_* = 10.4 Hz, 2F, Ar-**F**), -114.12 (t, *J_F-H_* = 9.8 Hz, 1F, Ar-**F**), -117.56 (t, *J_F-H_* = 9.4 Hz, 1F, Ar-**F**).

**Fig. S122**	^1^H NMR spectra of **NC0121** in CDCl_3_**.**

**Fig. S123**	^13^C{^1^H} NMR spectra of **NC0121** in CDCl_3_**.**

**Fig. S124**	^19^F NMR spectra of **NC0121** in CDCl_3_**.**

**NC0022**

*4'-cyano-[1,1'-biphenyl]-4-yl 2',3,5,6'-tetrafluoro-4'-propyl-[1,1'-biphenyl]-4-carboxylate*

Yield:	(white crystalline solid) 125 mg, 51 %

Re-crystallisation solvent:	EtOH

^1^H NMR (501 MHz):	7.77 – 7.68 (m, 4H, Ar-**H**)^*^, 7.66 (ddd, *J* = 8.8, 2.7, 2.1 Hz, 2H, Ar-**H**), 7.40 (ddd, *J* = 8.6, 2.7, 2.0 Hz, 2H, Ar-**H**), 7.19 (d, *J* = 8.9 Hz, 2H, Ar-**H**), 6.87 (ddd, *J* = 9.0, 3.0, 2.3 Hz, 2H, Ar-**H**), 2.63 (t, *J* = 6.7 Hz, 2H, Ar-C**H_2_**-CH_2_), 1.69 (h, *J* = 7.4 Hz, 2H, CH_2_-C**H_2_**-CH_3_), 0.99 (t, *J* = 7.4 Hz, 3H, CH_2_-C**H_3_**). ^*^Overlapping Signals.

^13^C{^1^H} NMR (126 MHz):	161.90 – 159.64 (m)^*^, 159.59 (dd, *J* = 250.5, 7.1 Hz), 150.93, 147.30 (t, *J* = 9.5 Hz), 144.83, 137.53, 135.99 (t, *J* = 11.3 Hz), 132.82, 128.63, 127.90, 122.47, 118.98, 114.49 (d, *J* = 23.1 Hz), 112.62 (t, *J* = 17.9 Hz), 112.11 (dd, *J* = 25.3, 5.1 Hz), 111.31, 109.38 (t, *J* = 17.1 Hz), 37.80, 23.98, 13.76.^*^ Overlapping Signals.

^19^F NMR (376 MHz):	-109.53 (d, *J_F-H_* = 9.9 Hz, 2F, Ar-**F**), -114.95 (d, *J_F-H_* = 9.6 Hz, 2F, Ar-**F**).

**Fig. S125**	^1^H NMR spectra of **NC0022** in CDCl_3_**.**

**Fig. S126**	^13^C{^1^H} NMR spectra of **NC0022** in CDCl_3_**.**

**Fig. S127**	^19^F NMR spectra of **NC0022** in CDCl_3_**.**

**NC1022**

*4'-cyano-3'-fluoro-[1,1'-biphenyl]-4-yl 2',3,5,6'-tetrafluoro-4'-propyl-[1,1'-biphenyl]-4-carboxylate*

Yield:	(white crystalline solid) 96 mg, 38 %

Re-crystallisation solvent:	EtOH

^1^H NMR (501 MHz):	7.71 (dd, *J* = 8.1, 6.6 Hz, 1H, Ar-**H**), 7.65 (ddd, *J* = 8.8, 2.6, 2.1 Hz, 2H, Ar-**H**), 7.49 (dd, *J* = 8.1, 1.7 Hz, 1H, Ar-**H**), 7.46 – 7.40 (m, 3H)^*^, 7.19 (d, *J* = 9.1 Hz, 2H, Ar-**H**), 6.87 (ddd, *J* = 9.0, 2.9, 2.1 Hz, 2H Ar-**H**), 2.63 (t, *J* = 6.8 Hz, 2H, Ar-C**H_2_**-CH_2_), 1.69 (h, *J* = 7.5 Hz, 2H, CH_2_-C**H_2_**-CH_3_), 0.99 (t, *J* = 7.3 Hz, 3H, CH_2_-C**H_3_**). ^*^Overlapping Signals.

^13^C{^1^H} NMR (126 MHz):	163.62 (d, *J* = 259.1 Hz), 160.75 (dd, *J* = 257.9, 6.2 Hz), 160.62, 159.70 (dd, *J* = 250.4, 7.3 Hz), 151.38, 147.68 (d, *J* = 7.9 Hz), 147.33 (t, *J* = 9.5 Hz), 136.34, 136.10 (t, *J* = 11.2 Hz), 134.00, 128.64, 123.57, 122.67, 115.02 (d, *J* = 20.3 Hz), 114.56 (dd, *J* = 23.6, 2.6 Hz), 114.13, 112.61 (t, *J* = 17.7 Hz), 112.12 (dd, *J* = 24.8, 4.5 Hz), 109.20 (t, *J* = 17.2 Hz), 100.24 (d, *J* = 15.5 Hz), 37.82, 23.99, 13.77.

^19^F NMR (376 MHz):	-105.87 (dd, *J_F-H_* = 10.2 Hz, *J_F-H_* = 6.7 Hz, 1F, Ar-**F**), -109.46 (d, *J_F-H_* = 10.3 Hz, 2F, Ar-**F**), -114.95 (d, *J_F-H_* = 9.6 Hz, 2F, Ar-**F**).

**Fig. S128**	^1^H NMR spectra of **NC1022** in CDCl_3_**.**

**Fig. S129**	^13^C{^1^H} NMR spectra of **NC1022** in CDCl_3_**.**

**Fig. S130**	^19^F NMR spectra of **NC1022** in CDCl_3_**.**

**4	Supplemental References**

1	Frisch, M. J. *et al*. Gaussian 16 Rev. C.01 (Wallingford, CT, 2016).

2	Becke, A. D. Density-functional thermochemistry. III. The role of exact exchange. J. Chem. Phys. 98, 5648 (1993).

3	Lee, C., Weitao, Y. & Parr, R. G. Development of the Colic-Salvetti correlation-energy formula into a functional of the electron density. Phys. Rev. B 37, 785 (1988).

4	Dunning, T. H., Jr. Gaussian basis sets for use in correlated molecular calculations. I. The atoms boron through neon and hydrogen. J. Chem. Phys. 90, 1007-1023 (1989).

5	Wang, J., Wolf, R. M., Caldwell, J. W., Kollman, P. A. & Case, D. A. Development and testing of a general amber force field. J. Comp. Chem. 25, 1157-1174 (2004).

6	Boyd, N. J. & Wilson, M. R. Validating an optimized GAFF force field for liquid crystals: TNI predictions for bent-core mesogens and the first atomistic predictions of a dark conglomerate phase. Phys. Chem. Chem. Phys. 20, 1485-1496 (2018).

7	Boyd, N. J. & Wilson, M. R. Optimization of the GAFF force field to describe liquid crystal molecules: the path to a dramatic improvement in transition temperature predictions. Phys. Chem. Chem. Phys. 17, 24851-24865 (2015).

8	Wang, J., Cieplak, P. & Kollman, P. A. How well does a restrained electrostatic potential (RESP) model perform in calculating conformational energies of organic and biological molecules? J. Comp. Chem. 21, 1049-1074 (2000).

9	Case, D. A. et al. AmberTools. J Chem. Info. Mod. 63, 6183-6191 (2023).

10	Sousa da Silva, A. W. & Vranken, W. F. ACPYPE - AnteChamber PYthon Parser interfacE. BMC Res. Notes 5, 367 (2012).

11	Abraham, M. J. et al. GROMACS: High performance molecular simulations through multi-level parallelism from laptops to supercomputers. SoftwareX 1-2, 19-25 (2015).

12	Hess, B., Bekker, H., Berendsen, H. J. C. & Fraaije, J. G. E. M. LINCS: A linear constraint solver for molecular simulations. J. Comp. Chem. 18, 1463-1472 (1997).

13	McGibbon, Robert T. et al. MDTraj: A Modern Open Library for the Analysis of Molecular Dynamics Trajectories. Biophys. J. 109, 1528-1532 (2015).

14	Mandle, R. J., Sebastián, N., Martinez-Perdiguero, J. & Mertelj, A. On the molecular origins of the ferroelectric splay nematic phase. Nat. Commun. 12, 4962 (2021).

15	Mandle, R. J. Implementation of a cylindrical distribution function for the analysis of anisotropic molecular dynamics simulations. PLOS ONE 17, e0279679 (2022).

16	Hobbs, J., Gibb, C. J. & Mandle, R. J. Emergent Antiferroelectric Ordering and the Coupling of Liquid Crystalline and Polar Order. Small Sci. 4, 2400189 (2024).

17	Hobbs, J. et al. Polar Order in a Fluid Like Ferroelectric with a Tilted Lamellar Structure – Observation of a Polar Smectic C (SmC_P_) Phase. Angew. Chem. In. Ed. 64, e202416545 (2025).

18	Gibb, C. J., Hobbs, J. & Mandle, R. J. Systematic Fluorination Is a Powerful Design Strategy toward Fluid Molecular Ferroelectrics. J. Am Chem. Soc. 147, 4571-4577 (2025).

19	Gibb, C. J. et al. Spontaneous symmetry breaking in polar fluids. Nat. Commun. 15, 5845 (2024).
